# Single-cell characterization of anti–LAG-3 and anti–PD-1 combination treatment in patients with melanoma

**DOI:** 10.1172/JCI164809

**Published:** 2023-03-15

**Authors:** Jani Huuhtanen, Henna Kasanen, Katriina Peltola, Tapio Lönnberg, Virpi Glumoff, Oscar Brück, Olli Dufva, Karita Peltonen, Johanna Vikkula, Emmi Jokinen, Mette Ilander, Moon Hee Lee, Siru Mäkelä, Marta Nyakas, Bin Li, Micaela Hernberg, Petri Bono, Harri Lähdesmäki, Anna Kreutzman, Satu Mustjoki

**Affiliations:** 1 Translational Immunology Research Program and Department of Clinical Chemistry and Hematology, University of Helsinki, Helsinki, Finland.; 2 Hematology Research Unit Helsinki, Helsinki University Hospital Comprehensive Cancer Center, Helsinki, Finland.; 3 Department of Computer Science, Aalto University, Espoo, Finland.; 4 iCAN Digital Precision Cancer Medicine Flagship, Helsinki, Finland.; 5 Department of Oncology, Helsinki University Hospital Comprehensive Cancer Center, Helsinki, Finland.; 6 Turku Bioscience Centre, University of Turku and Åbo Akademi University, Turku, Finland.; 7 Research Unit of Biomedicine, Medical Microbiology and Immunology, University of Oulu, Oulu, Finland.; 8 Oslo University Hospital-Radiumhospitalet, Oslo, Norway.; 9 Bristol Myers Squibb (BMS) Research and Development, Princeton, New Jersey, USA.

**Keywords:** Immunology, Oncology, NK cells, Skin cancer, T cells

## Abstract

**Background:**

Relatlimab plus nivolumab (anti–lymphocyte-activation gene 3 plus anti–programmed death 1 [anti–LAG-3+anti–PD-1]) has been approved by the FDA as a first-line therapy for stage III/IV melanoma, but its detailed effect on the immune system is unknown.

**Methods:**

We evaluated blood samples from 40 immunotherapy-naive or prior immunotherapy–refractory patients with metastatic melanoma treated with anti–LAG-3+anti–PD-1 in a phase I trial using single-cell RNA and T cell receptor sequencing (scRNA+TCRαβ-Seq) combined with other multiomics profiling.

**Results:**

The highest *LAG3* expression was noted in NK cells, Tregs, and CD8^+^ T cells, and these cell populations underwent the most significant changes during the treatment. Adaptive NK cells were enriched in responders and underwent profound transcriptomic changes during the therapy, resulting in an active phenotype. *LAG3*^+^ Tregs expanded, but based on the transcriptome profile, became metabolically silent during the treatment. Last, higher baseline TCR clonality was observed in responding patients, and their expanding CD8^+^ T cell clones gained a more cytotoxic and NK-like phenotype.

**Conclusion:**

Anti–LAG-3+anti–PD-1 therapy has profound effects on NK cells and Tregs in addition to CD8^+^ T cells.

**Trial registration:**

ClinicalTrials.gov (NCT01968109)

**Funding:**

Cancer Foundation Finland, Sigrid Juselius Foundation, Signe and Ane Gyllenberg Foundation, Relander Foundation, State funding for university-level health research in Finland, a Helsinki Institute of Life Sciences Fellow grant, Academy of Finland (grant numbers 314442, 311081, 335432, and 335436), and an investigator-initiated research grant from BMS.

## Graphical abstract

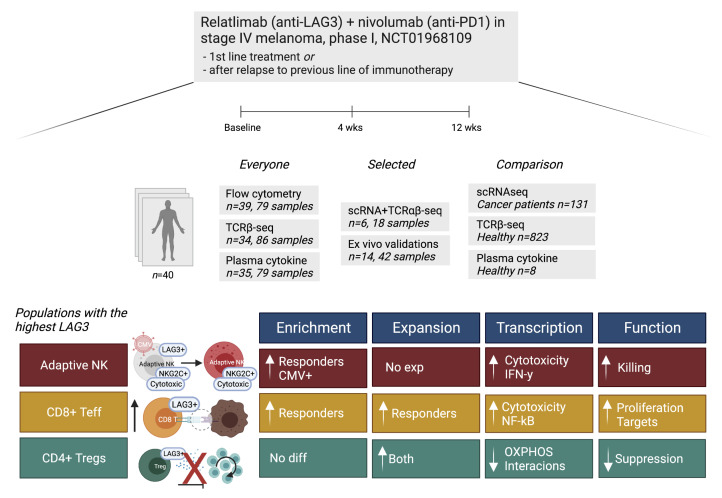

## Introduction

Even though immune checkpoint inhibitor therapies have revolutionized the treatment of metastatic melanoma, a majority of patients fail to achieve sustainable responses. As currently available immune checkpoint inhibitor therapies (anti–cytotoxic T lymphocyte–associated protein 4 [anti–CTLA-4], anti–programmed death 1 [anti–PD-1], and anti–programmed death ligand 1 [anti–PD-L1] therapies) primarily target effector CD8^+^ T cells, novel combination treatments that could also invigorate other immune cell types could increase the response rates in patients. Lymphocyte-activation gene 3 (LAG-3) is an inhibitory receptor expressed widely on different activated and exhausted immune cell subtypes (1–7), rendering it one of the most interesting novel immune checkpoint targets. Coinhibition of anti–LAG-3+anti–PD-1 is more attractive than blocking either LAG-3 or PD-1 alone (8), with encouraging efficacy even in patients with anti–PD-1/anti–PD-L1–refractory melanoma (9, 10). Relatlimab plus nivolumab (anti–LAG-3+anti–PD-1) combination therapy has shown a progression-free survival benefit over anti–PD-1 monotherapy as a first-line treatment for patients with metastatic melanoma (11) and has now been approved by the FDA. Although it is known that LAG-3 attenuates T cell activation, viability, and proliferation by binding to MHC class II molecules, knowledge of its effects on other immune cells is lagging (8, 12–15).

**Figure 1:**
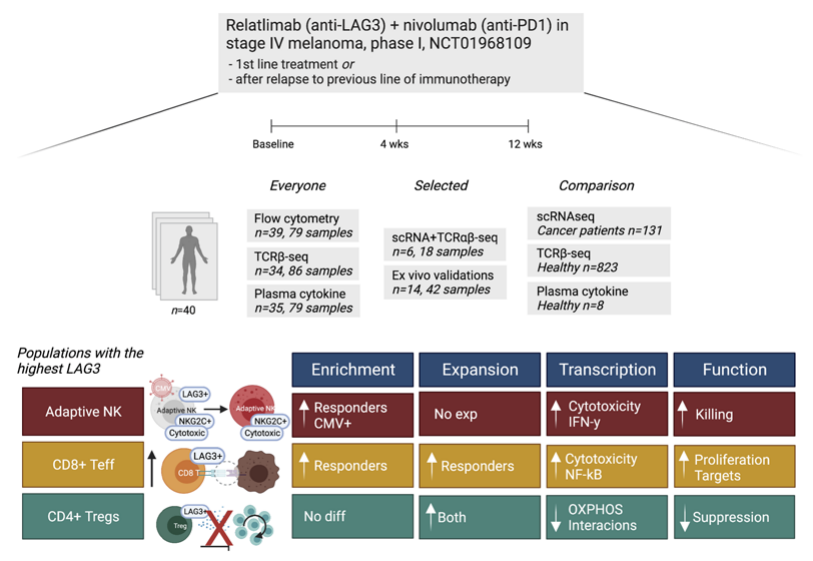
Single-cell profiling of anti-LAG3+anti–PD-1 treatment in patients with melanoma. Schematic of the study cohorts and main findings. diff, difference; exp, expansion. This figure was created with BioRender.com.

In this study, we used single-cell RNA and T cell receptor (TCR) sequencing (scRNA+TCRαβ-Seq), flow cytometry, TCRβ-Seq, and serum protein profiling together with ex vivo functional validations to analyze immune cell responses to anti–LAG-3+anti–PD-1 treatment (relatlimab+nivolumab, phase I, ClinicalTrials.gov NCT01968109) in pretreatment blood samples and blood samples taken 1 and 3 months after therapy from 40 patients with metastatic melanoma (Figure 1). The patients were either immunotherapy naive (IO naive) or prior immunotherapy refractory (IO refractory) (patient details are provided in Table 1 and Supplemental Table 1; supplemental material available online with this article; https://doi.org/10.1172/JCI164809DS1), and during the therapy more changes were observed in the immune cell repertoire of IO-naive patients. Anti–LAG-3*+*anti*–*PD-1 shifted LAG-3^+^CD8^+^ antigen-experienced T cells from an exhausted to a more cytotoxic phenotype. However, we observed the greatest effect in CMV seropositivity–associated cell populations, such as in adaptive NK cells, resulting in an activated phenotype. This was mainly observed in the responding patients, and they had higher numbers of adaptive NK cells, CMV seropositivity, and a costimulatory cytokine environment before initiation of the treatment. Our results provide an understanding of the effects of anti–LAG-3+anti–PD-1 combination treatment in vivo in patients and highlight previously overlooked subpopulations of cells as targets of immune checkpoint therapies.

## Results

### Adaptive NK cells and CD8^+^ T cells have the highest LAG3 expression and are more numerous in responders.

In total, we had 40 patients, 11 of whom were IO naive (7 of 11 [63.6%] were complete responders [CRs] or partial responders [PRs] and 4 [36.4%] had progressive disease [PD]) and 29 of whom were IO refractory (15 of 29 [51.7%] were CR/PR or had stable disease [SD] and 14 of 29 [48.3%] had PD). All the patients in the IO-naive cohort received 80+240 mg doses of relatlimab+nivolumab, while in the IO-refractory cohort 20 of 29 (68.9%) received 80+240 mg doses and 9 of 29 (31.0%) received 160+480 mg doses. The prior IO-refractory patients received previously anti–PD-1 therapy (22 of 29 [75.9%]) or anti–CTLA-4 and then anti–PD-1 (7 of 29 [24.1%]) (Table 1 and Supplemental Table 1).

**Figure 2:**
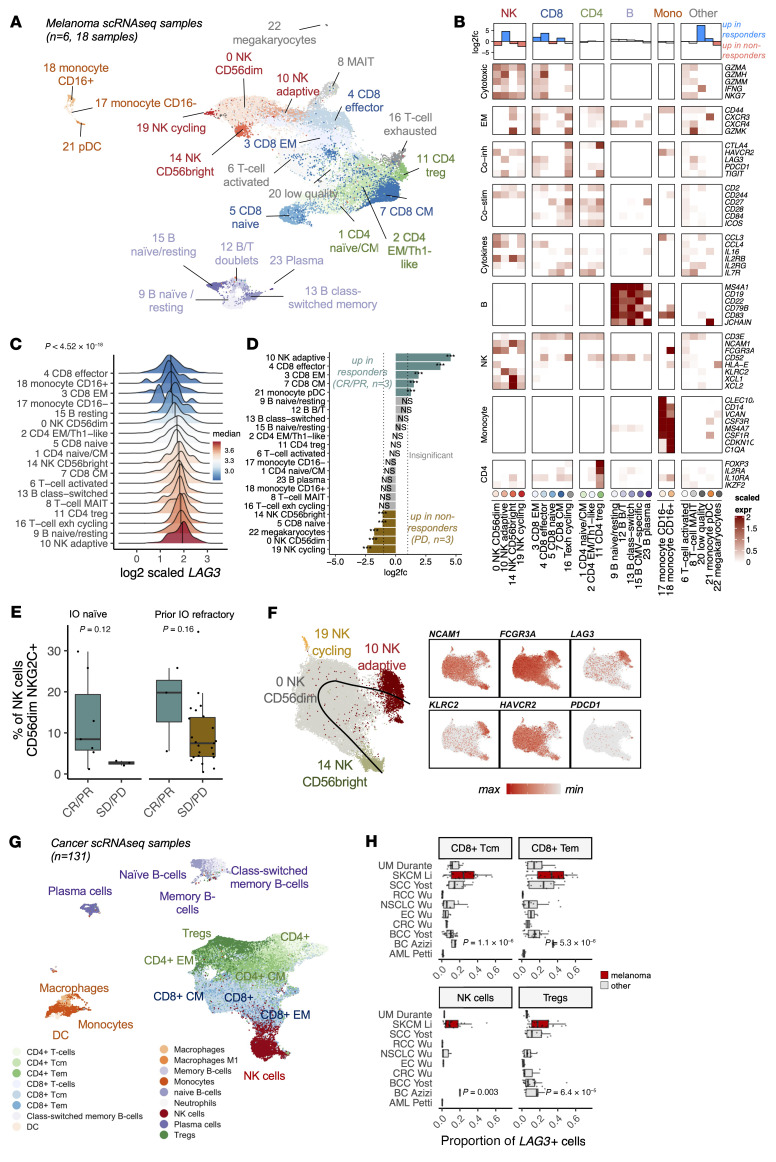
*LAG3* is expressed at high levels in Tregs and CMV-associated adaptive NK cells. (**A**) UMAP representation of CD45^+^-sorted cells in 18 scRNA+TCRαβ-Seq samples from 6 patients with melanoma before and after 4 weeks and 12 weeks of anti–LAG-3+anti–PD-1 treatment, profiled with scRNA+TCRαβ-Seq. (**B**) Scaled expression (expr) of selected differentially expressed markers (*P_adj_* < 0.05, Bonferroni-corrected *t* test) used to annotate clusters. The top row shows the log_2_ fold change (log2fc) of population abundances between patients with (CR/PR, *n* = 3) and without (PD, *n* = 3) a response at baseline. CM, central memory; Co-stim, costimulation; Co-inh, coinhibition; EM, effector memory; Mono, monocyte. (**C**) *LAG3* expression at baseline as scaled, log_2_(× + 1) transformed values. The adjusted *P* value (Bonferroni-corrected *t* test) indicates the difference between adaptive NK cells and the other cell types. exh, exhausted. (**D**) scRNA-Seq population abundances between patients with (CR/PR, *n* = 3) and without (PD, *n* = 3) a response at baseline. *P* values were calculated with a Fisher’s 2-sided exact test, and significant values needed to have at least a |log_2_ fold change| >1. ***P < 0.001. (**E**) Proportion of CD56^dim^NKG2C^+^ adaptive NK cells among NK cells in IO-naive (CR/PR *n* = 7; SD/PD *n* = 4) and IO-refractory (CR/PR *n* = 3; SD/PD *n* = 26) groups at baseline. *P* values were calculated with the 2-sided Mann-Whitney *U* test. (**F**) Focused UMAP of NK cells, where the superimposed line corresponds to the predicted pseudotime maturation trajectory and scaled expression of markers used to identify the subpopulations. max, maximum; min, minimum. (**G**) UMAP representation of cells from 131 scRNA-Seq tumor biopsies or bone marrow aspirate samples from 10 different cancers profiled with 10× technology. Annotation was done with SingleR. (**H**) Proportion of *LAG3^+^* cells across different cancers. *P* values were calculated with the Kruskal-Wallis test.

With scRNA+TCRαβ-Seq, we profiled 18 peripheral blood (PB) samples from 5 IO-naive and 1 IO-refractory patients with metastatic melanoma treated with anti–LAG-3+anti–PD-1 combination therapy (CRs *n* = 2, PRs *n* = 1, PD *n* = 3; patient details are provided in Supplemental Table 1). We identified 24 cell clusters in the scRNA-Seq data (Figure 2, A and B), all of which were present in every sample, but their abundances varied between patients and time points (Supplemental Figure 1, A–E).

Prior to anti–LAG-3+anti–PD-1 treatment, we found that *LAG3* was highly expressed in CD8^+^ T cells, CD4^+^ Tregs, and B cells, but the highest expression of *LAG3* was surprisingly detected in adaptive NK cells in the scRNA-Seq data (adjusted *P* value [*P_adj_*] < 0.0001, Bonferroni-corrected *t* test, Figure 2C), which was validated by flow cytometry (*n* = 8, Supplemental Figure 2, A and B). The largest difference in cell population abundances between patients with a response (CR/PR) and without a response (PD) was seen in adaptive NK cells in the scRNA-Seq data (*n =* 3 vs. *n =* 3, *P* < 0.0001, Fisher’s 2-sided exact test, Figure 2, B and D). This analysis was extended with flow cytometric data, in which we saw a similar, albeit not statistically significant, trend (CD56^dim^NKG2C^+^, IO naive *n =* 7 vs. *n =* 4 *P_adj_* > 0.05, prior IO refractory *n* = 3 vs. *n* = 26, Benjamini-Hochberg–corrected Mann-Whitney 2-sided *U* test, Figure 2E and Supplemental Table 1). Also, patients with a response had at least a 2-fold increase in the abundance of 3 non-naive CD8^+^ T cell clusters in the scRNA-Seq data (*P* < 0.0001, Fisher’s 2-sided exact test, Figure 2, B and D).

Adaptive NK cells, which were defined by the expression of *FCGR3A* (CD16a), *KLRC2* (NKG2C), and *ZEB2* as in the previous scRNA-Seq publications (16–18) and a lack of TCRs (Figure 2F and Supplemental Figure 2, C an D), share hallmarks of adaptive immunity with CD8^+^ T cells (19), including LAG-3–induced dysfunction (20). Adaptive NK cells are terminally mature NK cells (CD56^bright^ NK → CD56^dim^ NK → adaptive NK), and their maturation is accelerated by CMV infection (21). Accordingly, adaptive NK cells were found almost exclusively in CMV^+^ patients in the scRNA-Seq data (CMV^+^ *n* = 4, CMV^–^ *n* = 2, Supplemental Figure 2E) and in the more extensive flow cytometric data as well (CD56^dim^NKG2C^+^ [ref. 22], CMV^+^ *n* = 26, CMV^–^ *n* = 13, *P* < 0.01; Supplemental Figure 2F). CMV seropositivity was also associated with T cells with a NK-like phenotype in the flow cytometric data (CD4^+^CX3CR1^+^ and CD8^+^CX3CR1^+^, both *P* < 0.01, Supplemental Figure 2F) and increased T cell clonality in the TCRβ-Seq data (*P* < 0.01, Supplemental Figure 2G), both of which have previously been linked to immune checkpoint blockade responses (23–27).

### Immune cells in patients with melanoma have the highest expression of LAG3 in a pan-cancer analysis.

To validate *LAG3* expression levels in immune subsets in melanoma and to compare the levels with those in other human cancers, we pooled 131 tumor biopsy or bone marrow aspirate samples from 9 different cancers (acute myeloid leukemia [AML], breast cancer [BC], basal cell carcinoma [BCC], colorectal carcinoma [CRC], endometrial cancer [EC], non–small cell lung carcinoma [NSCLC], renal cell carcinoma [RCC], squamous cell carcinoma [SCC], skin cutaneous melanoma [SKCM], and uveal melanoma [UM]) profiled by similar scRNA-Seq methods (3, 28–33), together with deep generative modeling (34) and annotated tumor-infiltrating lymphocytes (TILs) with a cluster-agnostic approach (35) (Figure 2G and Supplemental Figure 3, A–F, cohorts in Supplemental Table 1). *LAG3* was confirmed to be highly expressed in tumor-infiltrating NK cells, Tregs, and B cell subsets in addition to CD8^+^ T cells (Supplemental Figure 3D), reflecting our data from PB. Across human cancers, melanoma samples exhibited the highest number of *LAG3*^+^ TILs, including *LAG3*^+^ NK cells, *LAG3*^+^ Tregs, and *LAG3*^+^CD8^+^ effector memory T cells (Figure 2H and Supplemental Figure 4, A and B). Importantly, *LAG3* expression was more abundant than *PDCD1* expression in all major TIL subsets in melanoma, unlike in other cancers (Supplemental Figure 4, C and D). These findings highlight the potential benefit of adding anti–LAG-3 to anti–PD-1 treatment, especially for patients with melanoma.

### Anti–LAG-3+anti–PD-1 treatment expands LAG3^+^ NK cells, CD8^+^ T cells, and CD4^+^ T cells in responding patients.

**Figure 3:**
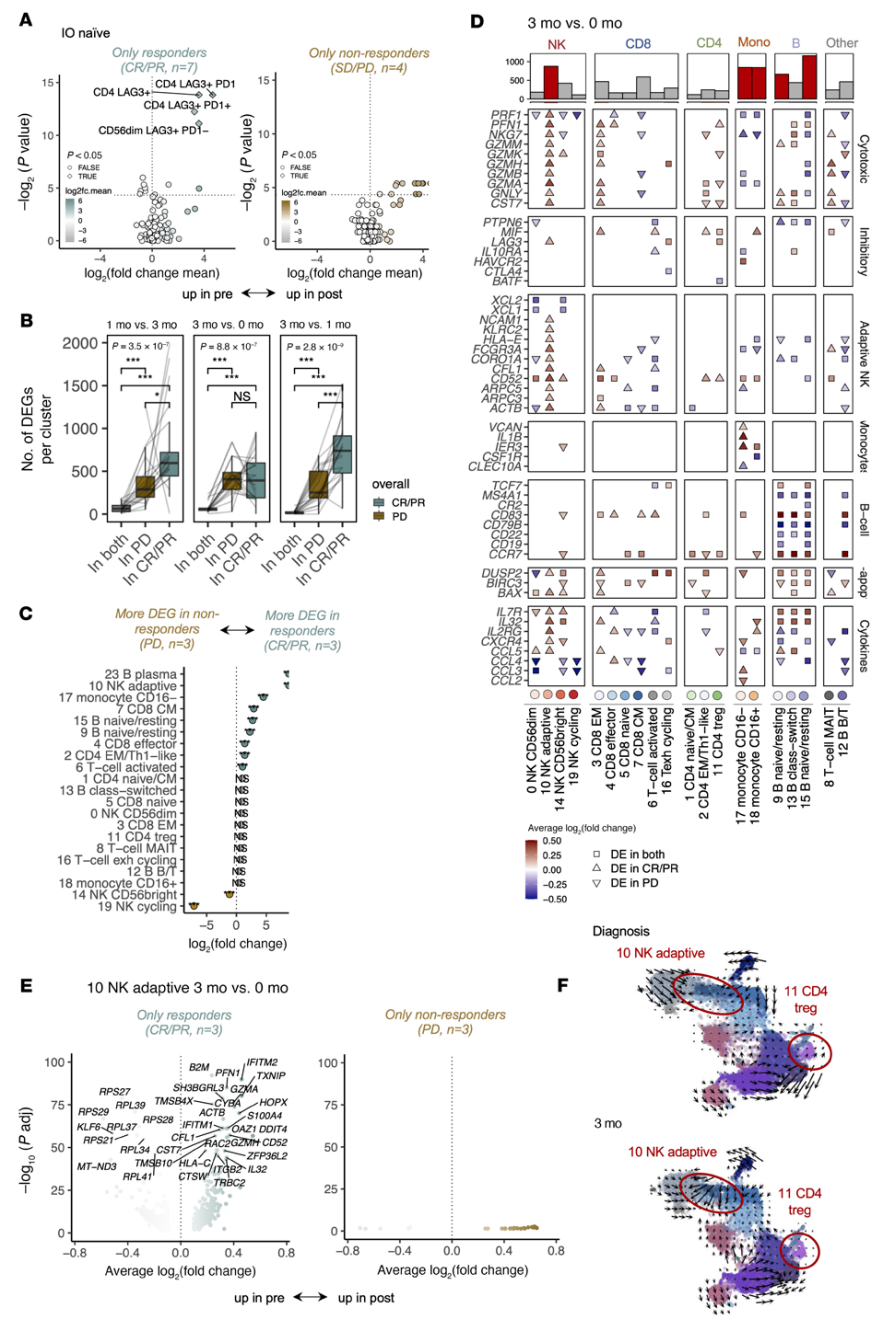
Anti–LAG-3+anti–PD-1 treatment upregulates *LAG3* and invigorates adaptive NK cells. (**A**) Differentially abundant (*P_adj_* < 0.05, Benjamini-Hochberg–corrected Mann-Whitney *U* test) flow cytometry subpopulations between 3 months of anti–LAG-3+anti–PD-1 treatment and baseline in IO-naive patients (*n* = 13) with a response (CR/PR *n* = 7) and with a nonresponse (SD/PD *n* = 4). The dashed line denotes *P* = 0.05. (**B**) The number of DEGs (*P_adj_* < 0.05, Bonferroni-corrected *t* test) between different time points in the different scRNA-Seq populations (Figure 2A) of cells from patients with (CR/PR, *n* = 3) or without (PD, *n* = 3) a response. *P* values were calculated with the Kruskal-Wallis test. (**C**) The log_2_ fold change of DEGs in scRNA-Seq data of patients with (CR/PR, *n* = 3) or without (PD, *n* = 3) a response after 1 month of anti–LAG-3+anti–PD-1 treatment. *P* values were calculated with Fisher’s 2-sided exact test, and significant values needed to have at least a |log_2_ fold change| >1. **P < 0.01 and ***P < 0.001. (**D**) DEGs in the scRNA-Seq data as log_2_ average fold changes (avg_logFC) between 3 months of anti–LAG-3+anti–PD-1 treatment and baseline. The shape denotes whether the gene was a DEG in patients with a response (DE in CR/PR), without a response (DE in PD), or in both (DE in both). Colors indicate upregulation at 3 months (red) or baseline (blue), and the shape indicates whether the DEG was found in patients with CR/PR, PD, or both. The bar plot on top shows the number of DEGs between 3 months of anti–LAG-3+anti–PD-1 treatment and baseline in different immune cell subpopulations, with the top 5 cell populations colored red. (**E**) DEGs (*P_adj_* < 0.05, Bonferroni-corrected *t* test) in adaptive NK cells (cluster 10) between 3 months (right) and baseline (left) in the scRNA-Seq data of patients with (CR/PR, *n* = 3) or without (PD, *n* = 3) a response. (**F**) UMAP representation of the scRNA-Seq samples from CMV-seropositive patients (*n* = 4), where superimposed arrows represent the directional flow calculated with Velocyto by comparing the abundances of spliced and unspliced mRNA reads. Arrows are smoothed with Gaussians.

Following anti–LAG-3+anti–PD-1 treatment, we noted statistically significant NK and T cell expansions in the flow cytometric data (*P_adj_* < 0.05, Benjamini-Hochberg–corrected, 2-sided Mann-Whitney *U* test) only in patients with a response (CR/PR in IO-naive patients *n* = 7, CR/PR/SD in prior IO–refractory patients *n =* 10), and no expansion in patients without a response (SD/PD in IO-naive patients *n* = 6, PD in prior IO–refractory patients *n* = 13, Figure 3A and Supplemental Figure 5, A–C; patient details and full results are provided in Supplemental Table 1). In the responders, *LAG3*^+^ lymphocyte expansion was noted in both the scRNA-Seq and flow cytometric data, including *LAG3*^+^CD4^+^ T cells in both IO-naive and prior IO–refractory patients, whereas expansion of *LAG3*^+^CD56^dim^ NK cells and *LAG3*^+^CD8^+^ T cells was only seen in the IO-naive cohort (Figure 3A and Supplemental Figure 5C). We found that cell populations coexpressing LAG-3 and PD-1 also expanded in the flow cytometric data, but unlike LAG-3^+^PD-1^–^ cells, the LAG-3^–^PD-1^+^ cells did not expand. These results hint that the therapy had a more noted effect on responding patients’ *LAG3*^+^ immune repertoire, especially in IO-naive patients. The different treatment doses of relatlimab+nivolumab (120+480 mg vs. 60+240 mg) did not result in differentially abundant cell populations (Supplemental Figure 5D).

### The phenotype of NK cells becomes active during anti–LAG-3+anti–PD-1 treatment in responding patients.

As therapies can have effects without causing population expansions, we calculated differentially expressed genes (DEGs) within subsets from the scRNA-Seq data (DEGs are listed in Supplemental Table 2). Responding patients (CR/PR, *n* = 3) had more notable transcriptomic changes (DEGs) already after 1 month of therapy in comparison with nonresponders (PD, *n* = 3) (*P* < 0.05, 2-sided Mann-Whitney *U* test, Figure 3B). At the 1-month point, the clusters with significantly more DEGs in responders in comparison with nonresponders included adaptive NK cells, CD8^+^ central memory T (Tcm) cells, and CD8^+^ effector T (Teff) cells (*P* < 0.001, Fisher’s exact 2-sided test, Figure 3C).

Although adaptive NK cells can effectively kill tumor cells (36), they have a limited proliferative capacity (37) and, hence unsurprisingly, did not expand following anti–LAG-3+anti–PD-1 therapy. Regardless, NK cells had the second highest number of DEGs, and in responders, the upregulated genes were associated with enhanced adaptive NK cell function (*FCGR3A* [CD16], *CD52*, *HLA-E*, *KLRC2*), cytotoxicity (*GZMA/B/H/K*, *GNLY,* *FGFBP2*, and *CST7*), and cytoskeletal remodeling to enable lytic granule secretion (*ACTB*, *ARPC3/5*, *CORO1A*, *CFL1*), as well as immediate early genes (*JUNB*, *NR4A2*) and antiapoptosis genes (*BAX*, *DUSP2*) in the scRNA-Seq data, and we did not observe these effects in nonresponders (Figure 3, D and E). The overall effect was that of upregulated pathways associated with the response to IFN-γ, which, together with the upregulated genes, indicate an active phenotype of these cells (Supplemental Figure 5E) (38). We tested this hypothesis in the scRNA-Seq data by studying the RNA turnover rate with RNA velocity (39), which showed that the previously quiescent adaptive NK cells initiated a strong directional flow after anti–LAG-3+anti–PD-1 therapy, suggesting elevated RNA transcription production previously not observed in these cells (Figure 3F and Supplemental Figure 6A).

### NK cells degranulate and secrete cytokines, and CD8^+^ T cells proliferate following anti–PD-1+anti–LAG-3^+^ therapy.

**Figure 4:**
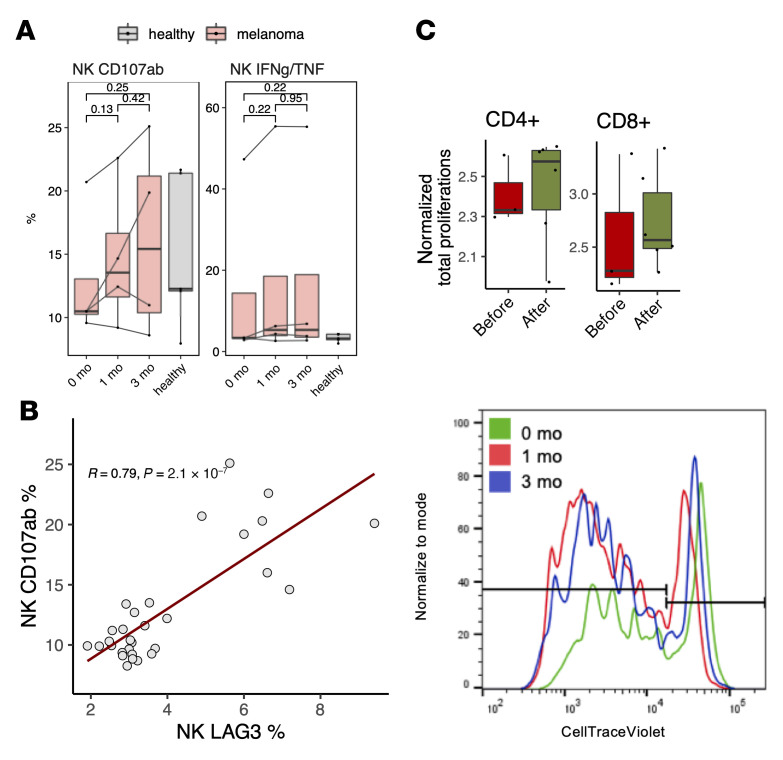
Anti–LAG-3+anti–PD-1 enhances NK cell degranulation, cytokine secretion, and T cell proliferation. (**A**) Degranulation (CD107a/b) and IFN-γ/TNF-α production of primary NK cells after stimulation with the chronic myelogenous leukemia (CML) cell line K562 in patients before (up in pre) and after (up in post) anti–LAG-3+anti–PD-1 treatment and in healthy donors at different time points (*n* = 4, 12 samples). When samples were available, they were examined as 3 replicates, but here, values are shown as averages. *P* values were calculated with the Kruskal-Wallis test. (**B**) Relationship between extracellular LAG-3 and degranulation responses (CD107a/b) to K562 target cells in melanoma samples before and after anti–LAG-3+anti–PD-1 treatment (*n* = 10, 30 samples). *P* values and correlation coefficients were calculated with Spearman’s rank correlation. (**C**) Top: Proliferation of CD4^+^ and CD8^+^ T cells induced by anti-CD3 and -CD28 beads (*n* = 3, 9 samples). Bottom: Cell divisions in CD4^+^ and CD8^+^ T cells after anti-CD3 and -CD28 bead stimulation in a selected patient. Cell proliferation was traced with flow cytometry by dilution of CellTrace Violet dye. Cells from different time points (before and after therapy) are marked with different colors.

To further validate the activated cell type, we performed ex vivo studies using the K562 cell line as a target for primary NK cells (Supplemental Figure 7A). In comparison with untreated samples, 3 of 4 post-therapy samples exhibited increased degranulation responses (CD107a/b) and elevated production of IFN-γ and TNF-α (Figure 4A), although the findings did not reach statistical significance due to the small number of samples. However, *TNF* was also one of the DEGs in the scRNA-Seq data for NK cells. Furthermore, LAG-3 expression correlated significantly with degranulation responses in NK cells (*P* < 0.0001, *R^2^* = 0.79, Spearman’s rank correlation), thus serving as a marker for elevated NK cell cytotoxicity (Figure 4B).

We observed upregulated expression of activation markers (*GZMA/H/K/M, GNLY, PRF1*) and downregulated expression of exhaustion markers (*CTLA4*) in responding patients’ CD8^+^ T cells in the scRNA-Seq data, and the most upregulated pathway was related to the NF-κB pathway and not the IFN-γ pathway (Supplemental Figure 5E). When stimulated with CD3/CD28 beads, we detected a trend toward higher T cell proliferation (including both CD4^+^ and CD8^+^ T cells) in ex vivo samples (*n* = 3) after anti–LAG-3+anti–PD-1 therapy compared with pre-therapy samples (Figure 4C). These findings were congruent with the flow cytometric data showing the expansion of LAG-3^+^CD4^+^ and LAG-3^+^CD8^+^ T cells in follow-up samples (Figure 3A and Supplemental Figure 5, A–C).

### Tregs expand in the periphery following anti–LAG-3+anti–PD-1 therapy.

**Figure 5:**
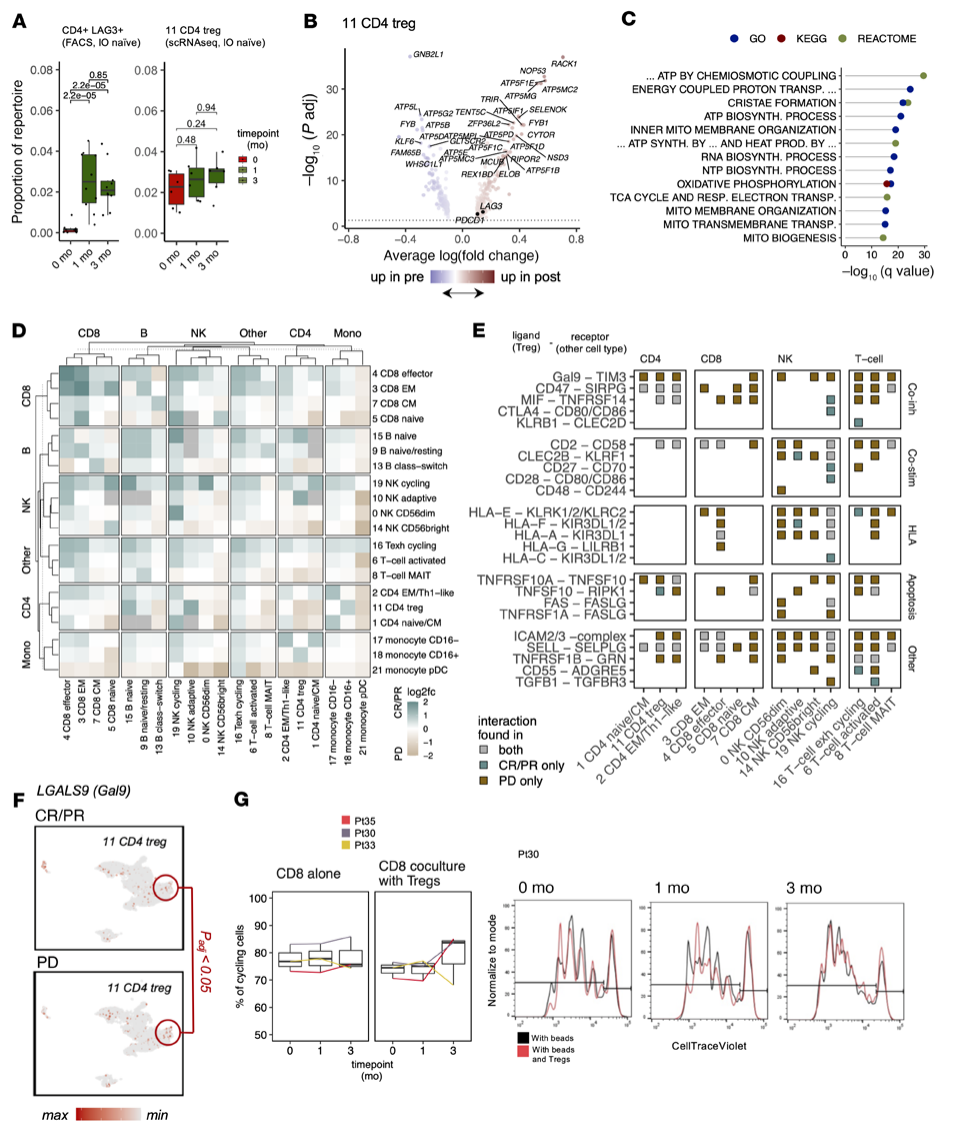
Anti–LAG-3+anti–PD-1 treatment expands peripheral Tregs but reduces their suppressive function. (**A**) Box plot showing the expansion of putative Tregs (CD4^+^LAG-3^+^) in flow cytometric data (left) for IO-naive patients (*n* = 11) and Tregs (cluster 11) in scRNA-Seq data (*n* = 6, right). *P* values were calculated with a 2-sided Mann-Whitney *U* test. (**B**) DEGs (*P_adj_* < 0.05, Bonferroni-corrected *t* test) in Tregs (cluster 11) between 1 month (right) and baseline (left) in the scRNA-Seq data. (**C**) The 15 most significantly downregulated pathways (*P_adj_* < 0.05, Benjamini-Hochberg corrected Fisher’s 2-sided exact test) in Tregs between 1 month and baseline in the scRNA-Seq data in different pathway databases (HALLMARK, GO, Reactome, and KEGG). Pathway names have been abbreviated for visualization purposes; full names are shown in Supplemental Table 2. (**D**) scRMA-Seq data–generated heatmap of the interactome in patients with (CR/PR, *n* = 3) or without (PD, *n* = 3) a response at baseline. The interactome is presented as a number of statistically significant ligand-receptor pairs (*P_adj_* < 0.05) calculated with CellPhoneDB. Green color indicates a higher number of interactions in patients with a response; beige color indicates a higher number of interactions in patients without a response. (**E**) Statistically significant inhibitory ligand-receptor interactions (*P_adj_* < 0.05, Benjamini-Hochberg–corrected CellPhoneDB permutation test) of Tregs with different immune cell subpopulations. The color denotes whether the interaction was exclusive in patients with (CR/PR) or without (PD) a response. (**F**) Expression of *LGALS9* (*Gal9*) in the scRNA-Seq of patients with (CR/PR, *n* = 3) or without (PD, *n* = 3) a response (Tregs are highlighted in red). (**G**) Suppression of CD8^+^ T cell proliferation by Tregs. Samples from 3 patients prior to and 1 month and 3 months after combination treatment were analyzed. The amount of cell proliferation was traced with flow cytometry by dilution of CellTrace Violet dye with the presence of CD3/CD28 beads and of CD3/CD28 beads and Tregs.

According to the flow cytometric data, LAG-3^+^CD4^+^ T cells were the most notably expanded cell population, in both IO-naive (*n* = 11) and prior IO–refractory patients (*n* = 29), especially in responding patients (Figure 3A, Supplemental Figure 5, A–D, and Supplemental Table 1). This population was already more abundant in the nonresponding IO-naive patients at baseline (Figure 2E). In the scRNA-Seq data, Tregs among the CD4^+^ T cell population showed the highest *LAG3* expression (Figure 2C). This CD4^+^LAG-3^+^ T cell population also expanded significantly following treatment in patients in the IO-naive cohort according to the flow cytometric data (Figure 5A). After therapy, scRNA-Seq data revealed upregulated expression in Tregs of *LAG3*, among other genes that are known to inhibit the proliferation and function of these cells (6) (Figure 5B and Supplemental Table 2). The top pathways lost in Tregs following anti–LAG-3+anti–PD-1 treatment in HALLMARK, Gene Ontology (GO), Reactome Pathway Database, and Kyoto Encyclopedia of Genes and Genomes (KEGG) categories included oxidative phosphorylation, the citric acid cycle, and ATP formation, suggesting decreased metabolic function (40) (Figure 5C, full pathways in Supplemental Table 2). This was also observed in a cell velocity analysis, in which Tregs appeared to adopt a more metabolically silent phenotype (Figure 3F).

As Tregs can inhibit immune cells via cell-cell contacts, we next sought to determine whether the observed changes resulted in alterations in predicted ligand-receptor interactions with CellPhoneDB (41) in the scRNA-Seq data (full ligand-receptor interactions are detailed in Supplemental Table 3). At baseline, the responders had more interactions than did nonresponders, most notably in different CD8^+^ T cell and NK cell populations (Figure 5D and Supplemental Figure 8A). The responders’ Tregs interacted more with adaptive NK cells, cycling NK cells, and exhausted T cells than did Tregs of nonresponders (Supplemental Figure 9A). The interactions seen with Tregs were mostly shared between both response groups, including different HLA interactions with NK cells, but the interactions between *LGALS9* (galectin 9) and its receptors (*HAVCR2*/*TIM-3*, *CD44*, and *CD47*) were exclusive to nonresponders (Figure 5E), and *LGALS9* was found to be upregulated in nonresponders’ Tregs (Figure 5F).

The cells that gained the most interactions in the scRNA-Seq data during the anti–LAG-3+anti–PD-1 therapy included adaptive NK cells, exhausted T cells, and different B cells (Supplemental Figure 9A). In contrast, Tregs were among the subsets that lost the most interactions, especially in responders (Supplemental Figure 9A). In the nonresponding patients, the interactions between Tregs and adaptive NK cells and CD8^+^ T effector cells increased (Supplemental Figure 9B). The interactions lost in responders included inhibitor interactions, such as *MIF*-*TNFRSF14*, *CTLA4*-*CD80/CD86*, and *KLRB1-CLEC2D*, which was not seen in the nonresponders (Supplemental Table 3).

To translate our findings to Treg phenotypes ex vivo, we performed coculture assays with primary Tregs and primary effector CD8^+^ or CD4^+^ T cells from the limited number of patient samples available (*n* = 3 at different time points). After 24 hours of coculturing, Tregs inhibited CD8^+^ and CD4^+^ Teff cell proliferation compared with controls without Tregs. Three months after the initiation of anti–LAG-3+anti–PD-1 therapy, the inhibitory effect of Tregs on CD8^+^ and CD4^+^ proliferation was slightly lower in 2 of the 3 pre-therapy samples, suggesting a decrease in Treg-suppressive function (Figure 5G and Supplemental Figure 9C).

### Anti–LAG-3+anti–PD-1 therapy increases chemotaxis and chemoattraction.

**Figure 6:**
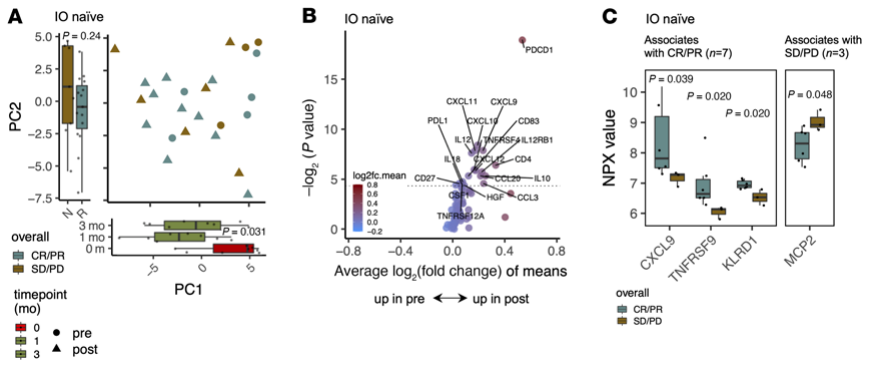
Cytokine profiling reveals increased chemotaxis and chemoattraction following anti–LAG-3+anti–PD-1 therapy. (**A**) PCA plot showing the serum protein profiles of individual samples from IO-naive samples (*n* = 11, R = CR/PR *n* = 7, N = SD/PD *n* = 4) at different time points, where the largest variation (PC1, 12.11%) is associated with before and after treatment, and the second-largest variation (PC2, 8.90%) is associated with an overall response. *P* values were calculated with the Kruskal-Wallis test. (**B**) Differentially expressed serum proteins (*P* < 0.05, 2-sided Mann-Whitney *U* test) between pre- and post-therapy samples in IO-naive patients (*n* = 11). No protein was upregulated at baseline. The dashed line denotes *P* = 0.05. (**C**) NPX values for all the soluble molecules associated with a response (CR/PR, *n* = 7) and without a response (SD/PD, *n* = 4) in the IO-naive patients. *P* values were calculated with the 2-sided Mann-Whitney *U* test.

As anti–LAG-3+anti–PD-1 treatment increased the number of cell-cell interactions in the scRNA-Seq data for most samples, we explored the cellular interactions further and profiled the levels of 78 different extracellular serum proteins (*n =* 35, 79 samples, Supplemental Table 1). Based on unsupervised principal component analysis (PCA) of the serum protein data, the largest variation (PC1, 12.11%) was between the pre– and post–anti–LAG-3+anti–PD-1 treatment samples (*P* < 0.05, Kruskal-Wallis test, Figure 6A). Following therapy, a greater number of cytokines were increased than decreased, especially in the IO-naive patients (*n* = 11, Figure 6B and Supplemental Figure 10, A–C); this was congruent with the increase in ligand-receptor pair predictions following therapy. The differences in cytokine environment were less prominent in the prior IO–refractory patients (*n* = 29, Supplemental Figure 10, A–C). The cytokines upregulated by the therapy hinted toward increased chemotaxis and chemoattraction for different leukocytes (CXCL9/-10/-11/-12, CCL3/-20), costimulating-enhancing molecules (CD27, TNFRSF4/OX40), IFN-γ production–enhancing molecules (IL-12/-18), and also antiinflammatory molecules (IL-10, PD-L1) (Figure 6B).

Before treatment initiation, the proteins that correlated with a favorable response included cytokines associated with a costimulatory environment, with upregulated CXCL9, TNFRSF9 (4-1BB), and KLRD1 (*P* < 0.05, 2-sided Mann-Whitney *U* test, Figure 6C), all implicated in favorable NK cell responses. The protein upregulated in nonresponding patients included only MCP2 (CCL8) (*P* < 0.05, Figure 6C), a known chemoattractant for myeloid cells.

### Higher T cell clonality in patients responding to anti–LAG-3+anti–PD-1 therapy.

**Figure 7:**
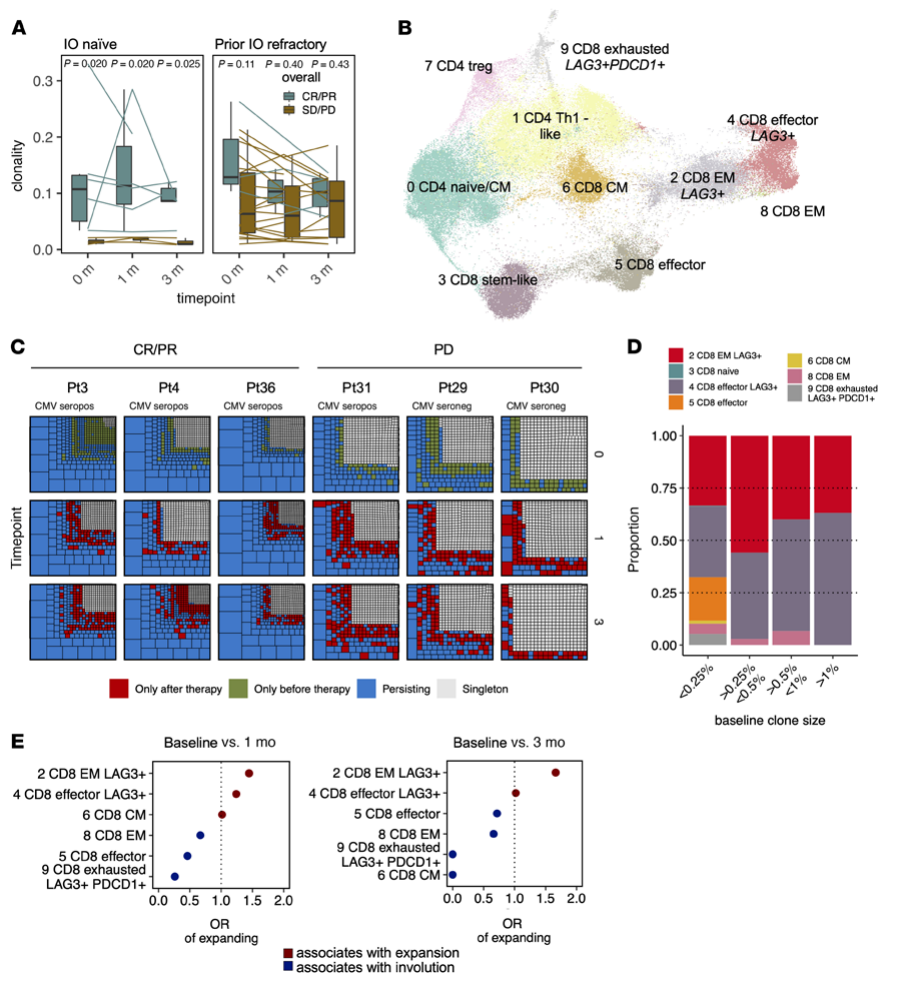
Higher baseline clonality is associated with a response to anti–LAG-3+anti–PD-1 therapy. (**A**) TCR repertoire clonality before and after anti–LAG-3+anti–PD-1 therapy in IO-naive patients (IO-naive *n* = 9, CR/PR *n* = 6, SD/PD *n* = 3, 26 samples) and IO-refractory patients (IO-refractory, *n* = 25, CR/PR *n* = 4, SD/PD *n* = 21, 44 samples). *P* values were calculated with a 2-sided Mann-Whitney *U* test. (**B**) UMAP representation of cells with detected TCRs from 18 scRNA+TCRαβ-Seq samples from patients with melanoma before treatment and 4 weeks and 12 weeks after treatment with anti–LAG-3+anti–PD-1 (*n* = 6, 18 samples), where the clusters are the same as in Figure 2A but renumbered based on size. (**C**) Treemap showing the clonal structure of the 500 most abundant clones from scRNA+TCRαβ-Seq–profiled patients, where each facet is a patient’s TCR repertoire at a different time point, and a box denotes a clonotype and the size of the box corresponds to the clonotype’s size. The boxes are colored on the basis of whether the clone is a singleton (gray, i.e., only 1 TCR read found) or persisting (blue; found before and following the therapy), novel (red; found only after the therapy), or contracting (green; found only before the therapy). The CMV serostatus of the patients is highlighted. seropos, seropositive; seroneg, seronegative. (**D**) The proportion of phenotypes of different clone size bins in pre-therapy samples. Different clones were assigned to different bins based on their size in the repertoire. (**E**) ORs for the expansion potential of clonotypes between 1 month after therapy and before therapy (baseline) and 3 months after therapy and before therapy (baseline), where higher ORs indicate a higher probability of expansion after therapy.

We analyzed TCRβ-Seq (anti–LAG-3+anti–PD-1–treated melanoma *n* = 34, 86 samples; healthy donors *n* = 783) and scRNA+TCRαβ-Seq (*n* = 6, 18 samples) data to understand the antigen restriction of expanded T cells. The baseline clonality was significantly higher in responding patients in the IO-naive cohort (*P* < 0.05, 2-sided Mann-Whitney *U* test) (Figure 7A and Supplemental Table 4). We also observed a similar trend in the prior IO–refractory cohort in responding patients at baseline, but it was not statistically significant (*P* > 0.05) (Figure 7A and Supplemental Table 4). In the prior IO–refractory cohort, the anti–LAG-3+anti–PD-1 treatment appeared to decrease overall blood T cell clonality in responding patients in the TCRβ-Seq data, although statistical significance was not reached.

### LAG3^+^CD8^+^ T cell clones expand following therapy and gain more cytotoxic and NK-like profiles in responding patients.

We linked the TCR information to T cell phenotype and noticed that the proportion of CD4^+^ and naive CD8^+^ T cells in the flow cytometric data correlated negatively with clonality in the TCRβ-Seq data, whereas the proportion of cytotoxic CD8^+^CD57^+^ T cells and CD56^dim^LAG3^+^PD-1^+^ NK cells were positively correlated (Supplemental Figure 11A). We also reclustered scRNA^+^ TCRαβ-Seq profiles of cells with detected TCRs (Figure 7B and Supplemental Figure 11, B and C). In the CMV^+^ patients (*n* = 4), we observed larger clones than in the CMV^–^ patients (*n* = 2), and the large clones frequently persisted following therapy, although novel clones also expanded (Figure 7C). The large clones (explaining at least >0.5 % of the repertoire) were of CD8^+^*LAG3*^+^ effector (cluster 4) and CD8^+^*LAG3*^+^ effector memory phenotypes (cluster 2) (Figure 7D). The clonotypes that were from these *LAG3*^+^ clusters 2 and 4 were the ones that were most likely to expand following therapy, both at the 1- and 3-month time points (Figure 7E).

**Figure 8:**
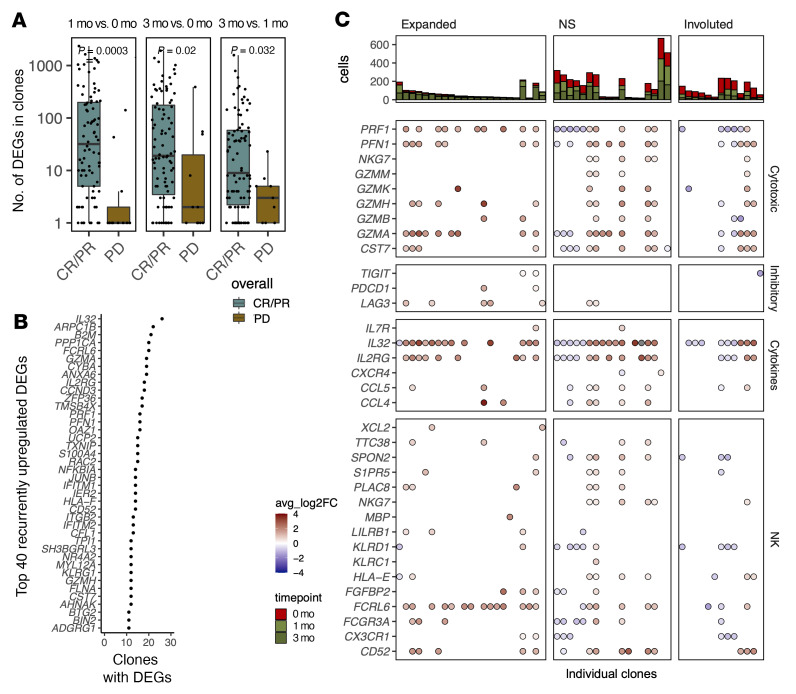
Clones from responding patients undergo more transcriptional alterations. (**A**) Number of DEGs (*P_adj_* < 0.05, Bonferroni-corrected *t* test) between different time points in the T cell clones (with at least 3 cells in each time point) from patients with (CR/PR, *n* = 3) and without (PD, *n* = 3) a response. *P* values were calculated with the 2-sided Mann-Whitney *U* test. (**B**) The top 40 most recurrently upregulated DEGs (*P_adj_* < 0.05, Bonferroni-corrected *t* test) in the clones from responders (CR/PR, *n* = 3). (**C**) Selected list of DEGs (*P_adj_* < 0.05, Bonferroni-corrected *t* test) in the scRNA-Seq data with log_2_ average fold changes between 3 months of anti–LAG-3+anti–PD-1 treatment and baseline in individual clonotypes. The facets are divided by whether the clone expanded over 2-fold, involuted over 2-fold, or persisted (NS) between the 2 time points. The bar plots on top show the number of cells in the clones at different time points.

We next analyzed the individual clonotypes in the scRNA+TCRαβ-Seq data and noticed that clones from responding patients had more transcriptomic alterations than did those from nonresponding patients at early and late response time points (1 month vs. baseline *P* < 0.0001, 3 month vs. baseline *P* < 0.05, 2-sided Mann-Whitney *U* test, Figure 8A). The most recurrently upregulated genes in the clones from responding patients included genes associated with cytotoxicity (*GZMA/H*, *PRF1*, *PNF1*, *S100A4*, *KLRG1*, *CST7*), cytokines (*IL32*, *IL2RG*), cell structure remodeling (*ADGRG1*, *ARPC1B*, *ANXA6*, *TMSB4X*, *FLNA*), class I HLA (*HLA-F*, *B2M*), and calcium signaling (*AHNAK1*, *S100A4*) (Figure 8B). When studying the clones that expanded over 2-fold, involuted over 2-fold, or persisted, we noticed major upregulation in cytotoxicity in the expanded clones but also to a great extent in the persisting clonotypes (Figure 8C). The increased expression of genes associated with the NK-like phenotype was more clearly observed in the expanded clones than in the persisting clones or involuted clones (Figure 8C).

### Conserved antigen targets for LAG-3^+^CD8^+^ T cell clones.

**Figure 9:**
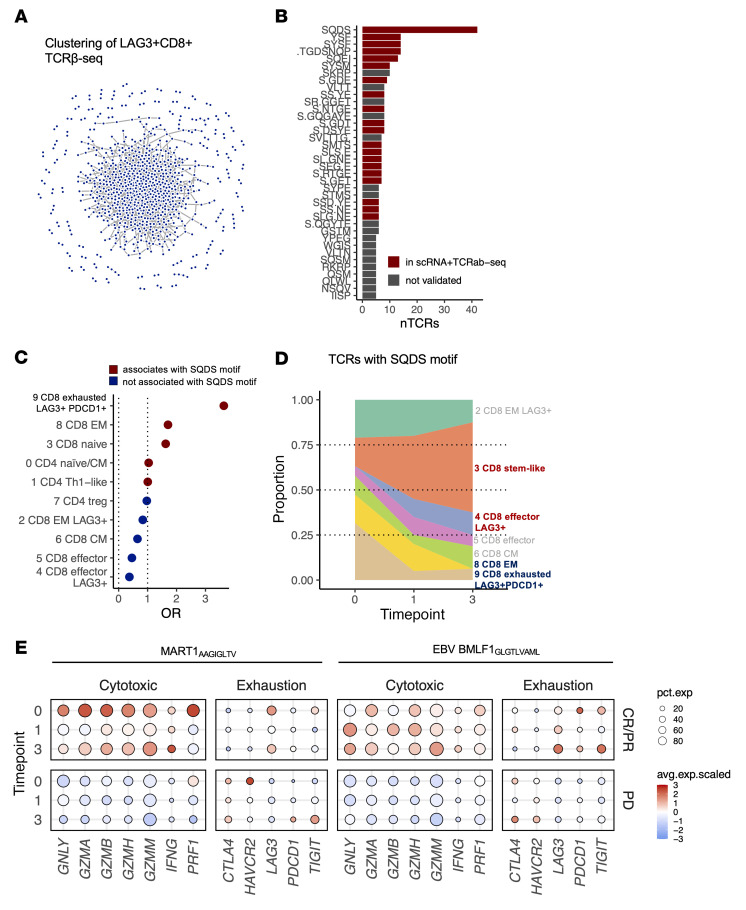
Anti–LAG-3+anti–PD-1 treatment invigorates LAG-3^+^CD8^+^ T cells targeting melanoma-associated antigens. (**A**) Network plot showing connections of similar TCRs in the LAG-3^+^CD8^+^–sorted TCRβ-Seq samples (*n* = 6), where a line between dots denotes amino acid–level similarities according to GLIPH2. (**B**) The number of clustered cells per TCR motif identified by GLIPH2 in the LAG-3^+^CD8^+^-sorted TCRβ-Seq samples, where the coloring indicates whether the motif was also identified in the analysis of TCRαβ-Seq data (*n* = 6, 18 samples). (**C**) ORs for TCRs with shared SQDS motifs showing a bias toward 9 CD8^+^ exhausted LAG3^+^PDCD1^+^ phenotypes. (**D**) A change in the phenotype of cells with the shared SQDS motif in their TCRs showed a decrease in the number of exhausted cells and an increase in cytotoxic cells in a responding patient. (**E**) Scaled average expression (avg.exp) and proportion of antigen-specific T cells (pct.exp) expressing canonical T cell markers in patients with CR/PR (*n* = 3) and PD (*n* = 3). Anti-MAA T cells were defined with TCRGP prediction against the MART1_AAGIGLTV_ target and against the antiviral EBV BMLF1_GLCTLVAML_ target.

Next, we sought to find the targets of the LAG-3^+^CD8^+^ T cell clones. We performed additional TCRβ-Seq on CD4^+^LAG-3^+^– or CD8^+^LAG-3^+^–sorted cells (*n* = 6) and noted their diversification following therapy (Supplemental Figure 11D), although it was insignificant. We performed clustering of TCRs to putative antigen-specific clusters with GLIPH2 (42) using CD8^+^LAG-3^+^–sorted cells and gained 38 antigen-specific groups of CD8^+^LAG-3^+^ T cells (Figure 9A). To determine whether these motifs are recurrent, we wanted to validate these motifs with orthogonal data and thus matched them back to our scRNA+TCRαβ-Seq data, which were profiled from different donors. We found 20 of 38 motifs in CD8^+^ T cells, with the most common motif being “SQDS” (Figure 9B). The phenotype of T cells with the same “SQDS” motif showed a bias toward the CD8^+^ exhausted phenotype, marked by expression of, e.g., *LAG3* and *PDCD1* (Figure 9C), meaning that the “SQDS” motif showed a similar phenotype in both the bulk TCRβ-Seq data from 1 set of donors and in the scRNA-TCRαβ-Seq data from another set of donors. After anti–LAG-3+anti–PD-1 therapy, the proportion of these exhausted cells was reduced, and the proportion of LAG-3^+^CD8^+^ Teff (cluster 4) and CD8^+^ T cells with stem-like properties (cluster 3), which have previously been associated with therapy response (43), was increased (Figure 9D).

### Immune checkpoint inhibitor therapies reverse the exhaustion of clonotypes recognizing melanoma-associated antigens in responders.

Finally, as we could link the found motifs to any antigen-specificities, we predicted the antigen specificities of scRNA+TCRαβ-Seq cells with TCRGP (44), our machine-learning method that predicts the probability for T cells to recognize epitopes with known TCR-epitope pairs. We predicted T cells targeting melanoma-associated antigens (MAAs) (e.g., MART1_AAGIGILTV_, MART1_ELAGIGILTV_) (45) and compared these with clones targeting antiviral epitopes (e.g., CMV, EBV, and influenza A).

We chose to focus on clonotypes predicted to target MART1_AAGIGILTV_ (16 clonotypes) and EBV BMLF1_GLGTLVAML_ (5 clonotypes) epitopes, as they were the most abundantly predicted targets in the cohort. In CR/PR patients, the anti-MAA T cells had higher expression of cytotoxicity-related genes throughout the treatment than did patients with PD (Figure 9E). Especially following therapy, *IFNG* production of the anti-MAA T cells was elevated in CR/PR patients, which was not seen in patients with PD. Also, in the CR/PR patients the antiviral T cells were more cytotoxic than those in patients with PD but less toxic than the anti-MAA T cells in the same patients (Figure 9E). Although none of the patients had known active viremia, the level of exhaustion was higher in the antiviral T cells than in the anti-MAA T cells, but this did not alter during the therapy.

## Discussion

Currently used immune checkpoint inhibitor therapies are primarily directed at invigorating cytotoxic T cells, but dual-checkpoint inhibition may allow the simultaneous activation of other important immune cell subpopulations to improve response rates. Our comprehensive multiomics data set demonstrates that *LAG3* is not only expressed in CD8^+^ T cells but that it is also highly expressed in NK cells and Tregs of patients with melanoma. Furthermore, our results illuminate how successful anti–LAG-3+anti–PD-1 therapy can have an impact on these cells by increasing the cytotoxic phenotype of NK cells, activating antigen-restricted T cells, and changing the expression profile of Tregs.

As NK cells have a high potential to kill tumor cells, enhancing their function could augment responses in tumors in which T cell responses are deficient, e.g., in tumors with a low number of neoantigens and low or no expression of class I HLA (46–48). In addition to classical inhibitory NK cell receptors (killer cell immunoglobulin-like receptors [KIRs], leukocyte immunoglobin-like receptors [LIRs], and NKG2A), recent studies have found that immune checkpoint receptors previously associated with T cells, such as PD1, HAVCR2, and LAG3, can drive NK cell dysfunction (49). Although the balance between immune cell activation and exhaustion is delicate, our results demonstrate how anti–LAG-3+anti–PD-1 therapy can stimulate NK cells, and especially in adaptive NK cells, we observed an increase in the IFN-γ response, upregulation of cytotoxicity-associated genes, and elevated degranulation and cytokine production. Importantly, in the patients who had an objective response (CR/PR), the adaptive NK cells were increased already at baseline, and they underwent significant transcriptional changes during the therapy. These results suggest that (adaptive) NK cells may participate in antitumor activities by directly killing tumor cells and/or modifying the cytokine environment to elicit T cell responses, but further ex vivo and in vivo studies are needed to understand the putative mechanisms in detail. Tregs can lead to the failure of immune checkpoint inhibitor therapy by reducing cytotoxic cell proliferation, suppressing immune cell–mediated lysis, and providing an unfavorable cytokine environment (50). In general, the expression of *LAG3* in Tregs has been linked to the suppression of Tregs (6). Hence, it was interesting to observe in the scRNA-Seq data that, following the anti–LAG-3+anti–PD-1 therapy, Tregs (and CD4^+^LAG-3^+^ cells, putative Tregs, in the flow cytometric analysis) expanded in both responding and nonresponding patients. Previous studies with anti–PD-1 have also detected a similar rise in Tregs in patients responding to anti–PD-1 treatment (43, 51, 52), which could be thought to prevent a prolonged, nonspecific immune reaction once the antitumor immune response has been activated. In the responding patients, anti–LAG-3+anti–PD-1 combination therapy decreased the number of predicted interactions between Tregs and other immune cells, which may affect their function and suppressive capacity. This is in accordance with the noted decrease in the suppressive capacity of CD4^+^ and CD8^+^ T cells by Tregs in the post-therapy samples, although the small number of samples available prevents strong conclusions. Interestingly, nonresponding patients had multiple *Gal9*-*TIM3* interactions between Tregs and effector cells, offering a hypothesis for a resistance mechanism for anti–LAG-3+anti–PD-1 therapy.

Prior studies have noted that patients responding to anti–PD-1 (with or without anti–CTLA-4) therapy have higher baseline TCR clonality in the tumor microenvironment (24) and harbor large clones that gain more DEGs during therapy than do nonresponding patients (23). Similarly, in our cohort, the patients responding to anti–LAG-3+anti–PD-1 therapy had higher baseline clonalities and larger clones that also had increased numbers of DEGs. However, our sample size was not sufficient large enough to determine whether the therapy significantly alters the clonality or diversity of the PB TCR repertoire. Anti–LAG-3+anti–PD-1 therapy also expanded CD8^+^LAG-3^+^ clones and shifted the phenotype of these clones to a more cytotoxic one with NK-like properties. We observed the highest increase in cytotoxicity in the clones that expanded, and rarely in involuting clones. Interestingly, we found the same TCR motifs in the CD8^+^LAG-3^+^ clones in different data sets, which could denote that cells with this phenotype have similar targets across patients, and anti–LAG-3+anti–PD-1 treatment may reduce the exhaustion of these antigen-specific T cells or prevent the precursor exhausted cells to become fully exhausted. Further studies with modern methods combining simultaneously scRNA+TCRαβ+peptide-major histocompatibility complex (pMHC) are needed to identify the specificity of these expanded T cell clones (53). However, we were able to obtain some evidence that anti–LAG-3+anti–PD-1 treatment can affect the phenotype of tumor-targeting T cell clones, as our supervised analysis with predicted anti-MART1_AAGIGILTV_ clones showed that during treatment, the phenotype of these cells changed from *LAG3*^+^ effector cells to cells with increased *IFNG* expression.

Our study has several potential limitations. As no tumor biopsies were available, the analysis was performed on immune cell subsets from PB samples and not from tumor samples, which could have been more informative for some aspects of the tumor reactivity. However, we used publicly available data on previously profiled baseline tumor samples to validate and extrapolate our results from PB samples. Also, the scRNA-Seq cohort was limited to 6 patients, the functional validations were done with a small number of patient samples, and no control arm with nivolumab alone was available in this phase I trial. Further studies are warranted to associate our findings in peripheral immune cell subsets with tumor samples from larger patient cohorts with comparison with other types of therapies in randomized phase II/III trials studying anti–LAG-3 combination therapies to reveal the detailed mechanism of action of this drug.

In summary, our study provides insights into anti–LAG-3+anti–PD-1 therapy in the human immune system and demonstrates the impact of the combination treatment on both NK cells and T cells.

## Methods

### Patients and samples.

This translational substudy includes 40 patients with metastatic melanoma, who were enrolled in the multicenter phase I trial (NCT01968109) (9) and treated with relatlimab (anti–LAG-3) in combination with nivolumab (anti–PD-1) according to the inclusion and exclusion criteria stated in the clinical trial protocol (protocol no. CA224020, BMS) at the Helsinki University Hospital Comprehensive Cancer Center in Finland or Oslo University Hospital in Norway. Given the phase I nature of the trial, no patients or investigators were blinded to the treatment. Of the 40 patients, 11 were IO naive, and 29 patients had been previously treated with either anti–PD-1 monotherapy or with anti–PD-1 in combination with anti-CTLA-4. One patient had also been treated with macrophage colony-stimulating factor 1 in combination with anti–PD-1. Clinical response data are presented as the best overall confirmed response per immune-related response criteria, with confirmation per Response Evaluation Criteria in Solid Tumors 1.1, according to the clinical study protocol. The database was locked in February in 2021. For detailed patient characteristics, see Supplemental Table 1. PB samples were obtained from the patients before initiation of the treatment at cycle 1, day 1 (C1D1), and follow-up samples were obtained during cycle 1, day 29 (C1D29) and cycle 2, day 1 (C2D1). PBMCs were separated using Ficoll-Paque density-gradient centrifugation (GE Healthcare) and were live-frozen at –150°C in 10% DMSO-FBS solution for further assays. Plasma was separated by centrifugation and then stored at –70°C.

### scRNA-Seq and analysis of the anti–LAG-3+anti–PD-1–treated cohort.

Single cells were partitioned using a Chromium Controller (10X Genomics), and scRNA-Seq and TCRαβ libraries were prepared using the Chromium Single Cell 5′ Library & Gel Bead Kit (10X Genomics), as per the manufacturer’s instructions (CG000086). In brief, approximately 17,000 cells from each sample, suspended in 0.04% BSA in PBS, were loaded onto the Chromium Single Cell A Chip. During the run, single-cell barcoded cDNA was generated in nanodroplet partitions. The droplets were subsequently reversed, and the remaining steps were performed in bulk. Full-length cDNA was amplified using 14 cycles of PCR (Veriti, Applied Biosystems). TCR cDNA was further amplified in a heminested PCR reaction using the Chromium Single Cell Human T Cell V(D)J Enrichment Kit (10X Genomics). Finally, total cDNA and TCR-enriched cDNA were subjected to fragmentation, end-repair and A-tailing, adaptor ligation, and sample index PCR (14 and 9 cycles, respectively). The gene expression libraries were sequenced using an Illumina NovaSeq, S1 flowcell with the following read length configuration: read 1 = 26, i7 = 8, i5 = 0, read 2 = 91. The TCR-enriched libraries were sequenced using an Illumina HiSeq 2500 in Rapid Run mode with the following read length configuration: read 1 = 150, i7 = 8, i5 = 0, read 2 = 150. The raw data were processed using Cell Ranger 3.0.0 with GRCh38 as the reference genome with default parameters.

All cells were subjected to quality control. Cells with high amounts of mitochondrial transcripts (>15% of all UMI counts) or ribosomal transcripts (>50%); cells with fewer than 100 genes or more than 4,500 genes expressed; cells expressing low or high (<25% or >60%) amounts of housekeeping genes; or cells with low or high read depths (<500 or >30,000 UMI counts) were excluded from the analyses.

To overcome the batch effect, we used a probabilistic framework to account for different nuisance factors of variation in an unsupervised manner with a deep generative modeling method scVI. Briefly, the transcriptome of each cell is encoded through a nonlinear transformation into a low-dimensional, batch-corrected, latent embedding. The scVI (0.5.0) algorithm (34) was ran with default parameters (n_hidden = 128, n_latent=30, n_layers =2, dispersion = ‘gene’) on all cells passing the quality control, with each sample treated as a separate batch. The latent embedding was then used for graph-based clustering implemented in Seurat (3.0.0) (54) and uniform manifold approximation and projection (UMAP) dimensionality reduction. For each different clustering, the genes related to V(D)J-recombination were removed, and the resolution values in FindClusters function were inspected visually within the range of 0.1–3 with intervals of 0.1, where the chosen values were within 0.2–0.5 to prevent overclustering. Clusters were named in descending order (cluster 0 contained the most cells) and annotated by analysis of canonical markers, DEGs, and relationships to other clusters in dimensionality-reduced plots, calculating different scores with predefined pathways used in previous publications (16, 17, 55, 56) and with the automated, reference-based cell annotation tool SingleR (1.2.4) (35), where Blueprint was used as a reference. For UMAP dimensionality reductions, the default parameters in RunUMAP function were used throughout. Pseudotime analyses were performed with Slingshot (version 1.1.4) (57) in unsupervised mode on precalculated UMAP coordinates with default parameters.

Different scores were calculated with Seurat’s AddModuleScore function, which is an implementation of the method suggested by Tirosh et al. (58). Differential expression analyses were performed on the basis of the *t* test, as suggested by Soneson and Robinson (59). Pathway analyses were conducted with the hypergeometric test on HALLMARK, GO, Reactome, or the KEGG categories with R package clusterProfiler (3.16.0) (60). The results are shown in Supplemental Table 2.

Heatmaps were generated with the ComplexHeatmap package (version 2.4.2), in which different clustering analyses were performed with Ward’s linkage with default parameters and the seed set at 123.

The abundances of spliced and unspliced reads (RNA velocity) were analyzed with dropEst (0.8.5) (61) and Velocyto (0.17.17) (39) with default parameters. After normalization, the data were smoothed using kNN-smoothed pooling (k = 500) on the PCA reduced space, while the high-dimensional velocity vectors were projected on the predefined UMAP embeddings. Receptor-ligand interactions were calculated with CellPhoneDB (version 2.0.0) (41) with default parameters on subsampled cells from each cell type to have an identical number of cells for each subtype (at least 50) with 1,000 iterations for the permutation testing. The costimulatory and coinhibitory receptor-ligand pairs were gathered from Dufva and Pölönen et al. (29). Pseudotime analyses were done with Slingshot (version 1.1.4) (57) in the unsupervised mode on precalculated UMAP coordinates with default parameters.

For scTCRαβ-Seq, only TCR productive full-length sequence information was considered, and all ambiguous cells with multiple TCRα and/or TCRβ chains were removed. Clones were defined as having the same CDR3 amino acid sequence in both TCRαβ chains, if available, or just in the TCRβ chain.

### Immunophenotyping with flow cytometry and analysis.

Different immune cell subpopulations were immunophenotyped with flow cytometry from fresh PB samples with 6 panels of different cell-surface markers, including the following immune checkpoint receptors and cytotoxicity and migration markers: CD3-PerCP-Cy5.5 (BD, catalog 332771), CD4-PE-Cy7 (BD, catalog 560649), CD45-APC-H7 (BD, catalog 560178), CD8-BV510 (BD, 563919), CD56-BV421 (BD, 562751), CXCR1-FITC (BioLegend, catalog 341606), CD16-PE (BD, 561313), TCR γδ-APC (BD, catalog 555718), PD1-FITC (BD, catalog 557860), LAG3-PE (BD, catalog 125209), ICOS-PE-Cy7 (eBioscience, catalog 25-9948-42), CTLA-4–APC (BD, catalog 560938), HLA-DR-BB515 (BD, catalog 560938), CD27-PE (BD, catalog 555441), CD25-PE-Cy7 (BD, catalog 561405), CD11b-APC (BD, catalog 550019), NKG2C-AF488 (R&D Systems, catalog FAB138G), CD161-PE (BD, catalog 556081), NKG2D-PE-Cy7 (BD, catalog 562365), NKG2A-APC (R&D Systems, catalog FAB1059A), DNAM-BB515 (BD, catalog 565152), CD57-PE (BD, catalog 560844), NKp46-PE-Cy7 (BD, catalog 562101), NKp30-AF647 (BD, 558408), CXCR3-AF488 (BD, catalog 561730), CCR7-PE (R&D Systems, catalog FAB197P), CD45RO-PE-Cy7 (BD, catalog 560608), and CXCR4-APC (BD, catalog 560936). CD45^+^ lymphocytes were acquired with the BD FACS Verse, and the data were analyzed with FlowJo, version 10.4. The results are shown in Supplemental Table 1.

### Serum protein analysis with a multiplex immunoassay.

Serum samples separated from fresh PB using centrifugation were analyzed with a proximity extension assay (Proseek Multiplex Inflammation panel, Olink Bioscience). The samples were run on 2 separate plates, and duplicate samples were used to normalize the differences between the 2 runs. Protein levels were expressed as normalized protein expression (NPX) values, an arbitrary log_2_ scale unit. The results are shown in Supplemental Table 1.

### NK cytokine secretion and CD107a/b degranulation assay.

To study cytokine secretion and CD107a/b degranulation of LAG-3–expressing NK cells, previously frozen PBMCs from 4 patients at 0-, 1-, and 3-month time points and from 5 healthy controls were thawed in warm RPMI) media and allowed to rest overnight at 37°C 5% CO_2_ before stimulation with K562 cells for 6 hours at 37°C in 5% CO_2_. Anti–CD107a-FITC (BD, catalog 555800) and anti–CD107b-FITC (BD, catalog 555804) were used to measure degranulation. Calcium ionophore (MilliporeSigma, catalog C9275-1MG) and PMA (Cell Signaling Technology, catalog 4174S) stimulation was used as a positive control, and no stimulation was used for the negative control.

After the stimulation, the cells were washed once with PBS and stained with the following membrane-antibody mixes: CD3-PerCP-Cy5.5 (BD, catalog 332771), CD8-PE-Cy7 (BD, catalog 335822), CD45-APC-H7 (BD, catalog 560178), CD56-PE (BD, catalog 345812), and LAG3-APC (BioLegend, catalog 369212). After staining, the cells fixed and permeabilized with the Cytofix/Cytoperm kit (BD, catalog 554714) according to the manufacturer’s instructions and stained with an intracellular antibody mix of IFN-γ–BV450 (BD, catalog 560371), TNF-α–BV450 (BD, catalog 561311), and GZMB-BV510 (BD, catalog 563388) cytokines. A total of 150,000 cells were acquired with FACS Verse (BD), and the data were analyzed with FlowJo, version 10.4. The gating of cell populations is shown in Supplemental Figure 7A.

### Treg proliferation and suppression test.

CD3 cells were isolated from 10 million previously frozen PBMCs using the EasySep Human T Cell Isolation Kit (STEMCELL Technologies). The isolated CD3 cells were stained with antibodies CD3-AF488 (BD, catalog 557694), CD4-PerCP (BD, catalog 345770), CD25-APC (BD, catalog 555434), and CD127-PE (BD, catalog 557938) and sorted with the BD FACSAria III cell sorter to isolate CD8 (CD3^+^CD4^–^) and CD4 (CD4^+^CD127^+^) effector cells and Tregs (CD4^+^CD127^–^CD25^hi^).

The sorted CD4^+^ or CD8^+^ T cells were stained with the CellTrace Violet Cell Proliferation Kit (Thermo Fisher Scientific) according to the manufacturer’s protocol. Labeled effector cells (10,000 cells/well) were seeded in a 96-well, U-bottomed plate. To induce proliferation, the Gibco Dynabeads Human T Activator CD3/CD28 kit (Thermo Fisher Scientific) was used at a 1:20 Teff cell ratio. The proliferation of Teff cells was carried out for 72 hours before 10,000 freshly isolated Tregs were added either for 24 or 48 hours. To measure the proliferation after 96 or 120 hours of incubation, the cells were stained with a CD4-PE (BD, catalog 561841) and CD8-APC (BD, catalog 561953) antibody mix. The suppressive capacity of Tregs was calculated by comparing the division of Teff cells in the presence of Tregs with the control wells without Tregs.

### TCRβ-Seq and analysis.

TCRβ-Seq was conducted as previously described with the ImmunoSEQ assay by Adaptive Biotechnologies. Genomic DNA was used in all cases.

Analyses started with the TCRβ matrices provided by Adaptive Biotechnologies preprocessing pipeline. All data were transformed to the VDJtools (62) format to reduce the complexity of the data. Nonproductive clonotypes were removed from the analysis. To assess the saturation of the sequencing results between the cohorts, the dependencies between sample diversity and sample size were determined with rarefaction plots inspired by Colwell et al. (63) and implemented in VDJtools. We used a minimum sampling depth of 20,000 reads per sample and subsampled all samples with more reads to 20,000 reads to normalize the samples and remove biases for depth-dependent statistics. Multiple different diversity metrics, including Shannon-Wiener, Simpson, and clonality indexes, were calculated with the CalcDiversityStats function on both unsampled and subsampled repertoire data (see Supplemental Table 3).

To identify T cells with the same epitope specificities, we used the online server for GLIPH 2 (42), which groups TCRs that potentially recognize the same target by calculating global and local amino acid similarities and compares these clusters with clusters found from a reference set of TCRs from naive, singleton T cells to determine the statistical significance with default parameters.

Epitope specificity predictions were performed with TCRGP (version 1.0.0), and anti-MAA models (MART1_AAGIGILTV_, MART1_ELAGIGILTV_, MELOE1_TLNDECWPA_, TKT_AMFWSVPTV_, and SEC24A_FLYNLLTRV_) were gathered from the Huuhtanen et al. report (45). The antiviral models (influenza A M1 _GILGFVFTL_, EBV BMLF1_GLCTLVAML_, CMV pp65_IPSINVHHY_, CMV pp65_NLVPMVATV_, EBV BZLF1_RAKFKQLL_, B7_RPRGEVRFL_, CMV pp65_TPRVTGGGAM_, and EBV BRLF1_YVLDHLIVV_ epitope) were gathered from the TCRGP (44) package’s GitHub page (https://github.com/emmijokinen/TCRGP; commit ID e3c8e5c). For the predictions used in all analyses, a threshold corresponding to a false-positive rate (FPR) of 5% was determined for each epitope separately from the ROC curves obtained from the cross-validation experiments in the original publications.

### Data and code availability.

Patient characteristics, flow cytometry–profiled cell population abundances, and serum cytokine assay results are provided in Supplemental Table 1. The processed and raw single-cell data can be downloaded from the European Genome-Phenome Archive (EGA) (data set ID: EGAS00001005580). The preprocessed scRNA-Seq counts, scTCRαβ-Seq clonotypes, TCRβ-Seq clonotypes, and Seurat objects are available at Zenodo (https://doi.org/10.5281/zenodo.5747250). The processed scRNA+TCR-Seq data are available in ArrayExpress (E-MTAB-12733) and TCR-Seq data are in immuneAccess (https://clients.adaptivebiotech.com/pub/huuhtanen-2023-jci) The TCRβ-Seq results are also shown in Supplemental Table 3. All the custom scripts to reproduce the key findings can be found in github.com/janihuuh/lag3_manuscript (commit ID a60e65a).

### Statistics.

*P* values were calculated with nonparametric tests, including the Mann-Whitney *U* test (2 groups), the Kruskal-Wallis test (more than 2 groups), and Fisher’s exact test where the alternative hypotheses are reported. Adjustments for multiple testing were performed when the number of tests exceeded 20 and were either done with Benjamini-Hochberg correction or Bonferroni correction in the DEG analyses. Nominal *P* values and adjusted *P* values of less than 0.05 were considered significant. All calculations were done with R (version 4.0.2) or Python (version 3.7.4). In the box plots, the center line corresponds to the median, the box corresponds to the IQR, and the whiskers are 1.5 × the IQR, while outlier points are plotted individually where present.

### Study approval.

All patients and healthy controls gave their written informed consent. The study was approved by the Helsinki University Central Hospital (HUCH) ethics committee (Dnro 115/13/03/02/15) and was conducted in accordance with the Declaration of Helsinki.

## Author contributions

The study was conceived by JH, HK, AK, and S Mustjoki. The patients were recruited by MH, K Peltola, S Mäkelä, MN, HK, and PB. The clinical data were collected by OB. The clinical trial–associated data were provided by BL. scRNA-Seq was carried out by TL and HK with the help of JH. Flow cytometry was performed by AK and HK with help from MI, K Peltonen, and MHL. TCRβ-Seq and serum protein profiling were carried out by HK. Ex vivo functional validations were done by HK and VG. All the data analyses were designed and performed by JH with the help of OD, who assisted with interpreting the single-cell clusters, EJ, who helped with the TCR analyses, and JV, who assisted with the RNA velocity analyses. The comparison data to support the findings were acquired by JH. JH wrote the manuscript and produced the figures with the help of S Mustjoki and with contributions from all authors. The project was supervised by S Mustjoki with the help of AK and HL. The order of the co–first authors was determined on the basis of their contributions to the manuscript. All authors read and approved the final manuscript.

## Supplementary Material

Supplemental data

ICMJE disclosure forms

Supplemental table 1

Supplemental table 2

Supplemental table 3

Supplemental table 4

## Figures and Tables

**Table 1:**
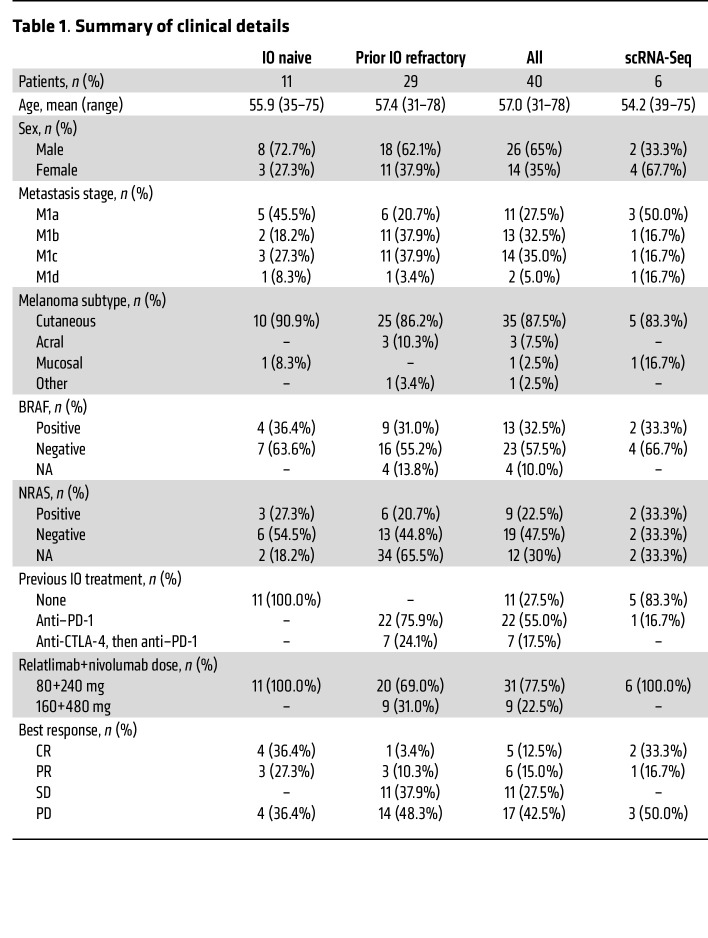
Summary of clinical details

## Version 1 01/31/2023

In-Press Preview

## Version 2 03/15/2023

Electronic publication

## Version 3 03/29/2023

Updated Acknowledgments
