# Supplementary material for: Single-cell characterization of anti–LAG-3 and anti–PD-1 combination treatment in patients with melanoma
Source: J Clin Invest. 2023 Mar 15;133(6):e164809. doi: 10.1172/JCI164809 (PMC10014104; doi:10.1172/JCI164809)
Supplement: Supplemental data [file jci-133-164809-s011.pdf]

Supplementary Material

Supplemental Figure 1: Annotation of clusters in anti-LAG3+anti-PD1 treated patients' peripheral blood
Supplemental Figure 2: LAG3 expression, annotation, and CMV-association of adaptive NK-cells
Supplemental Figure 3: Pan-cancer scRNAseq analysis of inhibitory receptor expression across immune subsets
Supplemental Figure 4: scRNA-seq analysis of inhibitory receptor expression across immune subsets in metastatic melanoma biopsies
Supplemental Figure 5: Immune subset abundance changes induced by anti-LAG3+anti-PD1 treatment
Supplemental Figure 6: The effect of anti-LAG3+anti-PD1 treatment on NK-cells in *ex vivo* analysis
Supplemental Figure 7: Changes in immune cell interactions induced by anti-LAG3+anti-PD1 treatment
Supplemental Figure 8: Serum protein changes induced by anti-LAG3+anti-PD1 treatment
Supplemental Figure 9: T-cell receptor repertoire analysis

Supplemental Table 1: Patient characteristics, flow cytometry population adundancies, and serum cytokine measurements
Supplemental Table 2: Differentially expressed genes and pathways from scRNA-seq data
Supplemental Table 3: Ligand-receptor analysis results
Supplemental Table 4: T-cell receptor repertoire analysis results

Supplemental Figure 1

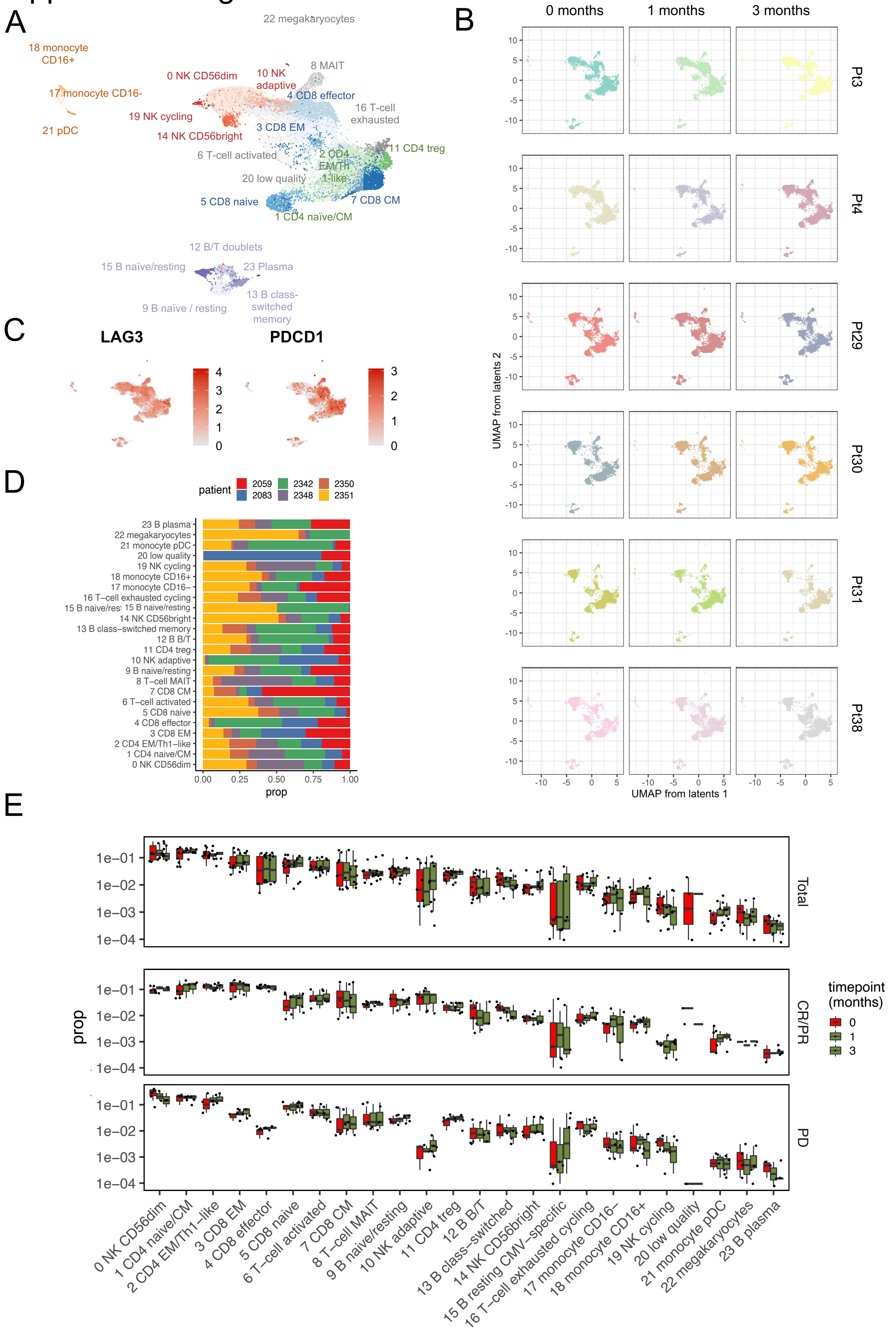

Supplemental Figure 1: Annotation of clusters in anti-LAG3+anti-PD1 treated patients' peripheral blood

A) The same UMAP representation as in Figure 2A showing CD45+ sorted cells from 18 scRNA+TCRαβ-seq samples from immunotherapy naïve from patients with metastatic melanoma from before, after 4 weeks, and after 12 weeks of anti-LAG3+anti-PD1 treatment profiled with scRNA+TCRαβ-seq.

B) Scaled expression of *LAG3* in and *PDCD1* in the UMAP space shown in panel A.

C) The distribution of different patients across different clusters in the UMAP space shown in panel A.

D) The distribution of cells from different samples in the UMAP space shown in panel A. Four first digits correspond to patient ID and the last digit corresponds to sampling time point.

E) The proportion of scRNAseq clusters across anti-LAG3+anti-PD1 treatment in total patients (*n*=6), patients with response (CR/PR, *n*=3), or patients without response (PD, *n*=3).

Supplemental Figure 2

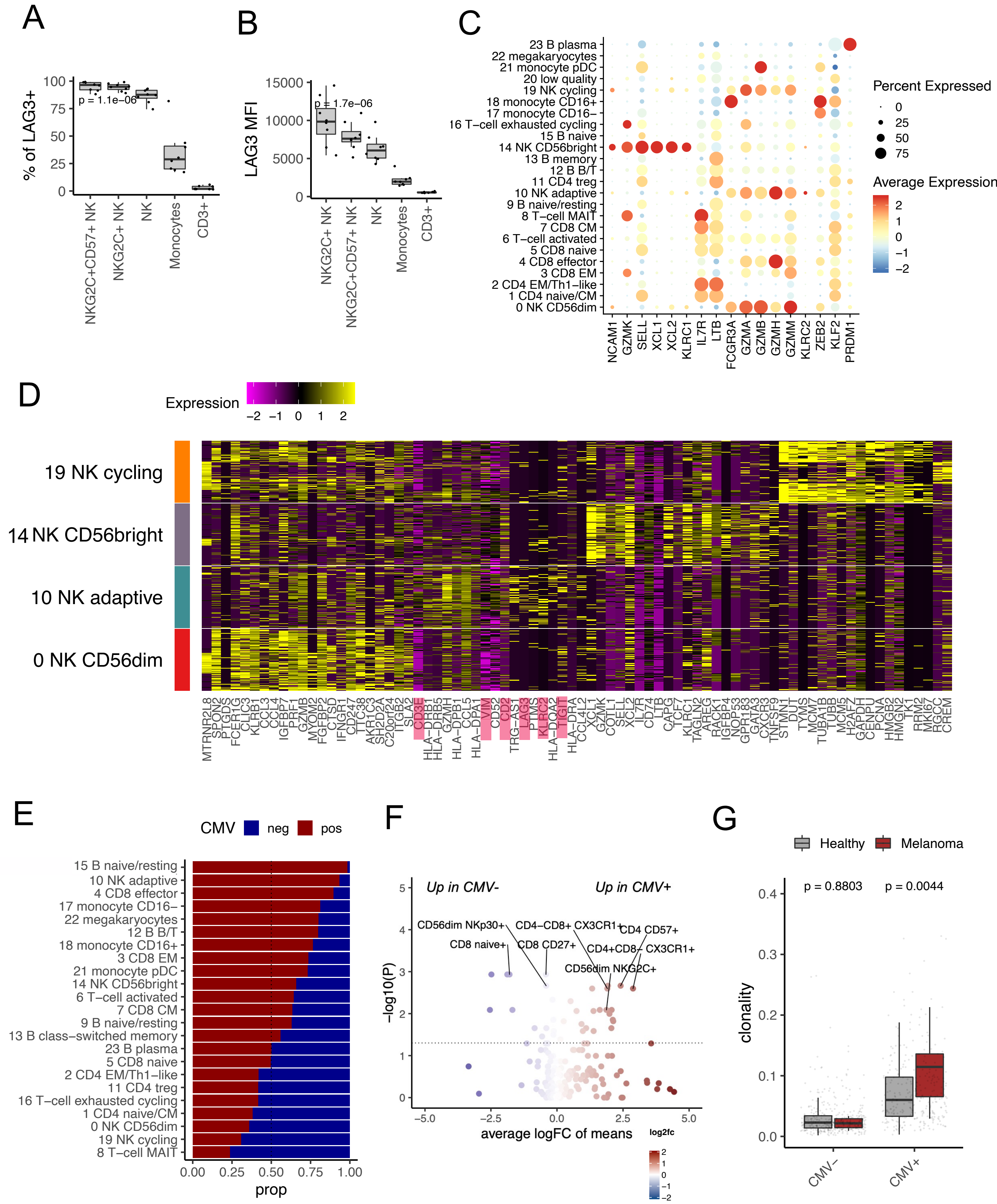

Supplemental Figure 2: LAG3 expression, annotation, and CMV-association of adaptive NK-cells

A) The abundancy of LAG3+ cells in different immune cell populations measured with flow cytometry in samples from patients with melanoma (*n*=8). *P*-value was calculated with a Kruskal-Wallis test.

B) The LAG3 expression as a mean fluorescent intensity (MFI) in different immune cell populations from patients with melanoma (*n*=8). *P*-value was calculated with a Kruskal-Wallis test.

C) Expression of a set of canonical markers used to identify adaptive NK cells as in previous scRNAseq publications. Dot size corresponds to the percentage of cells expressing a given gene in a given cluster and dot color corresponds to the average expression of a given gene in a given cluster. The clusters are the same as shown in Figure 2A.

D) The expression of the 25 most differentially expressed genes based on fold-change between NK-cell clusters shown as Z-values. Different inhibitory genes and markers associated with adaptive NK cells are highlighted.

E) The distribution of CMV seropositive (*n*=4) and seronegative (*n*=2) patients across different clusters.

F) Differential (P_{adj}<0.05, Benjamini-Hochberg corrected Mann-Whitney test) flow cytometry subpopulation abundances between CMV-seropositive (CMV+, n=26) and - seronegative (CMV-, *n*=13) patients in baseline samples from the anti-LAG3+anti-PD1 cohort. The dashed line denotes *P*=0.05.

G) The TCRβ-repertoire clonality in healthy donors (*n*=640) from Emerson et al., or patients with melanoma (*n*=27) from the present study, divided by CMV serostatus. *P*-values were calculated with a Mann-Whitney test.

Supplemental Figure 3

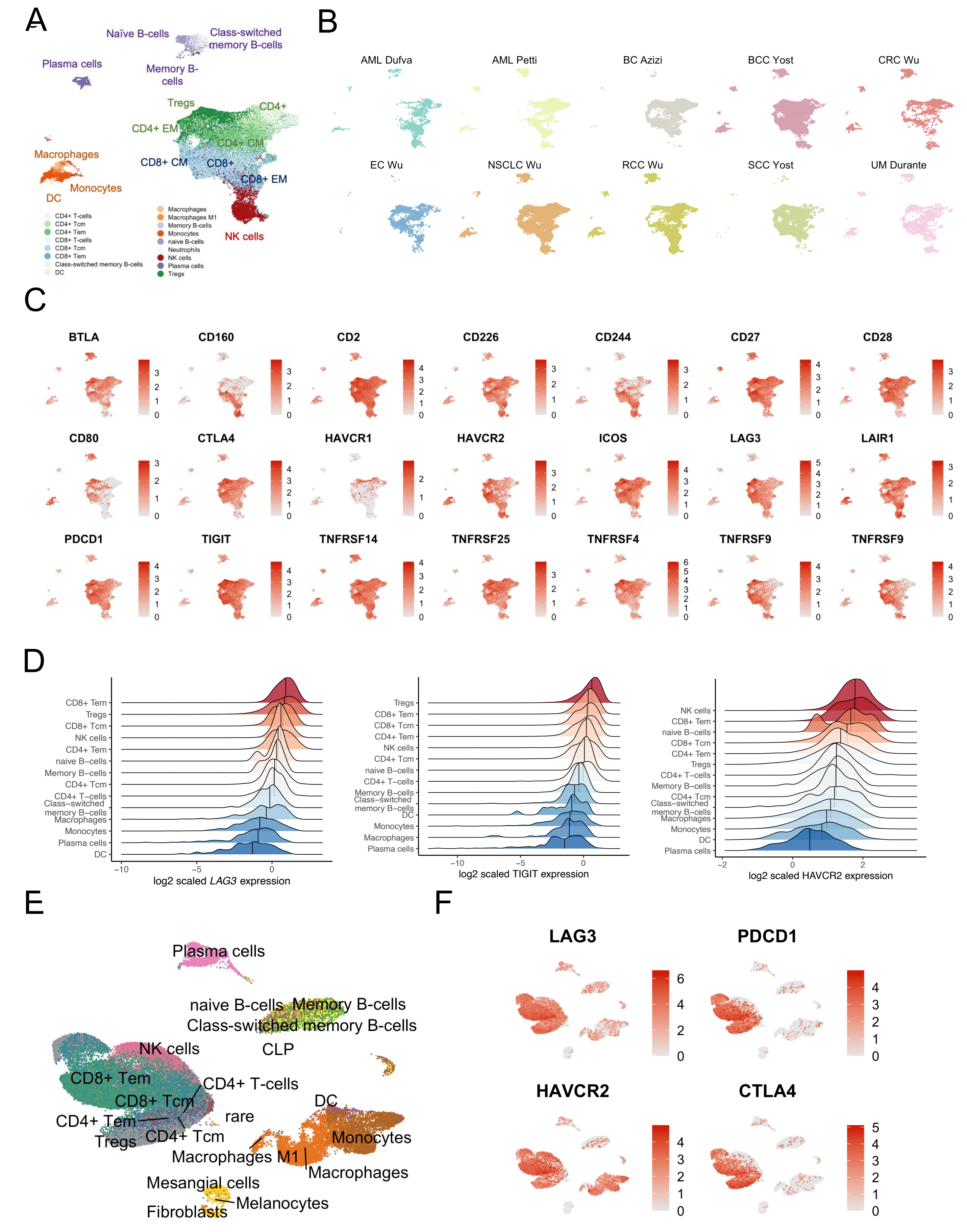

Supplemental Figure 3: Pan-cancer scRNAseq analysis of inhibitory receptor expression across immune subsets

A) The same UMAP representation as in Figure 2G showing cells from 131 scRNAseq biopsy or bone marrow aspirate samples from 10 different cancers profiled with droplet-based 10X technologies. Different cell types were annotated with reference-based method SingleR.

B) The distribution of cells from different cancer samples in the UMAP space. AML=acute myeloid leukemia, BC=breast cancer, BCC=basal cell carcinoma, CRC=colorectal carcinoma, EC=endometrial cancer, NSCLC=non-small cell lung carcinoma, RCC=renal cell carcinoma, SCC=squamous cell carcinoma, SKCM=skin cutaneous melanoma, UM=uveal melanoma.

C) The scaled expression of different inhibitory receptors in the UMAP space.

D) Expression of *LAG3, HAVCR2/TIM3,* and *TIGIT* in different immune subsets across different cancers shown as scaled, log2(x+1) transformed values.

E) UMAP representation of cells from 24 scRNAseq biopsies from patients with melanoma profiled with droplet-based DropSeq-technology. Different cell types were annotated with reference-based method SingleR.

F) The scaled expression of different inhibitory receptors in the UMAP space.

Supplemental Figure 4

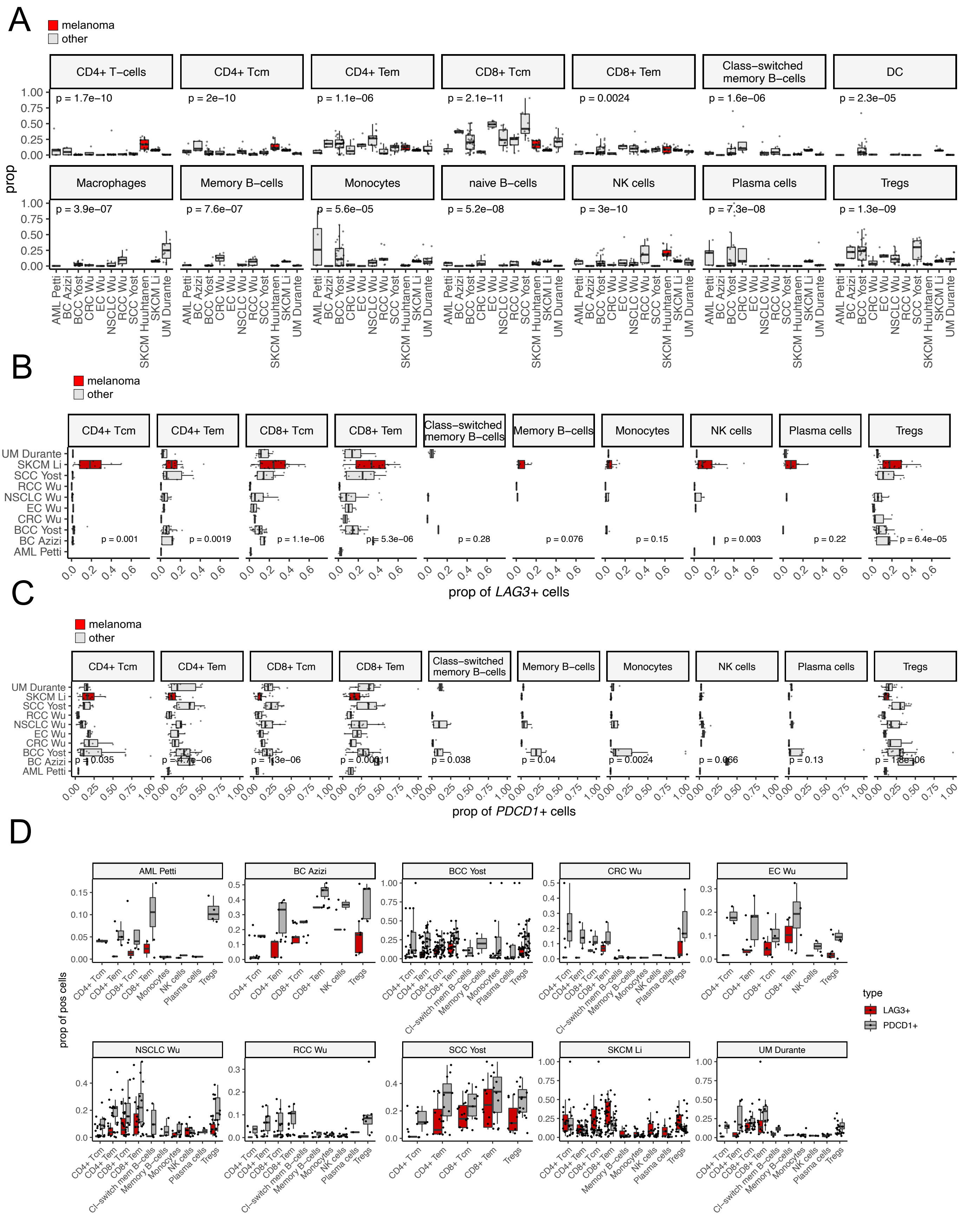

Supplemental Figure 5: scRNA-seq analysis of inhibitory receptor expression across immune subsets in metastatic melanoma biopsies

A) The proportion of different immune subsets identified by SingleR in individual samples across different cancers in the pancancer analysis shown in Figure 2G. *P*-values were calculated with a Kruskal-Wallis test. AML=acute myeloid leukemia, BC=breast cancer, BCC=basal cell carcinoma, CRC=colorectal carcinoma, EC=endometrial cancer, NSCLC=non-small cell lung carcinoma, RCC=renal cell carcinoma, SCC=squamous cell carcinoma, SKCM=skin cutaneous melanoma, UM=uveal melanoma.

B) The proportion of *LAG3*+ cells in different immune subsets across different cancers. *LAG3*+ was identified from the scRNA-seq data in a fashion similar to flow cytometry analysis, where any cell with noted *LAG3* expression was defined as positive. *P*-values were calculated with a Kruskal-Wallis test.

C) The proportion of *PDCD1*+ cells in different immune subsets across different cancers, where *PDCD1*+ was defined as in panel B. *P*-values were calculated with a Kruskal-Wallis test.

D) The proportion of *LAG3*+ and *PDCD1*+ cells in different immune subsets across different cancers, defined as in panel B and C.

Supplemental Figure 5

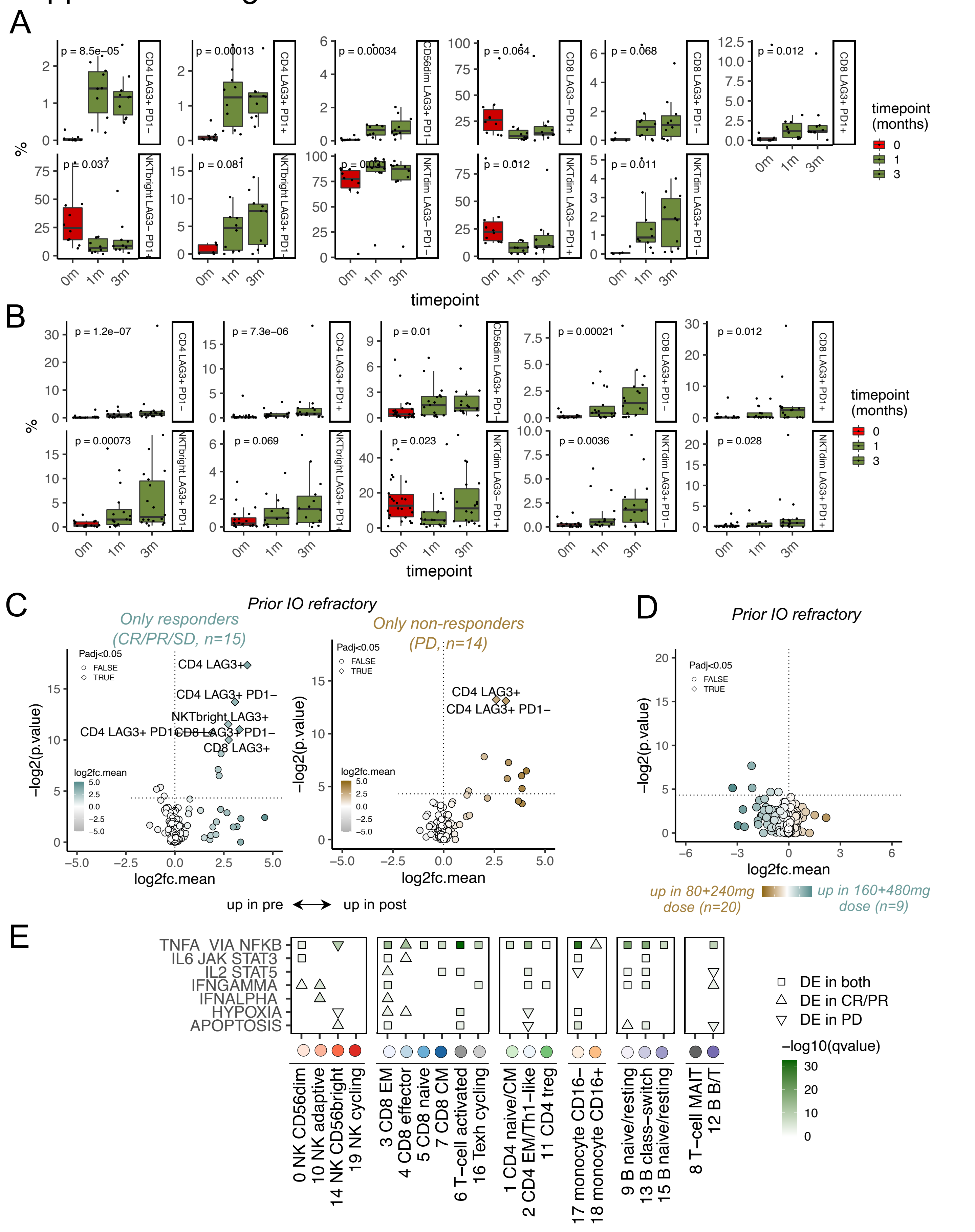

Supplemental Figure 5: Immune subset abundance changes induced by anti-LAG3+anti-PD1 treatment

A) The proportion of statistically significant (P<0.05, two-sided Mann-Whitney test) flow cytometry identified immune subsets across anti-LAG3+anti-PD1 treatment in immunotherapy (IO) naïve patients (n=11). P-values were calculated with Kruskal-Wallis test.

B) The proportion of statistically significant (P<0.05, two-sided Mann-Whitney test) flow cytometry identified immune subsets across anti-LAG3+anti-PD1 treatment in prior IO refractory patients (n=29). P-values were calculated with Kruskal-Wallis test.

C) Differentially abundant (P_{adj}<0.05, Benjamini-Hochberg corrected Mann-Whitney test) flow cytometry subpopulations between 3 months of anti-LAG3+anti-PD1 treatment and baseline in prior IO refractory patients with response (CR/PR/SD, n=15) or without (PD, n=14). The dashed line denotes P=0.05.

D) Differentially abundant (P_{adj}<0.05, Benjamini-Hochberg corrected Mann-Whitney test) flow cytometry subpopulations between reltalimab+nivolumab doses of 160+480mg and 80+240mg after 3 months of anti-LAG3+anti-PD1 treatment in immunotherapy. The dashed line denotes P=0.05.

E) Heat map showing significantly upregulated recurrent (Benjamini-Hochberg adjusted Fisher's two-sided exact test Q<0.05 at least in 2 population) HALLMARK pathways as the -log10 of adjusted P-value. The shape denotes whether the pathway was a significant in patients with a response (CR/PR), without a response (PD), or in both.

Supplemental Figure 6

A

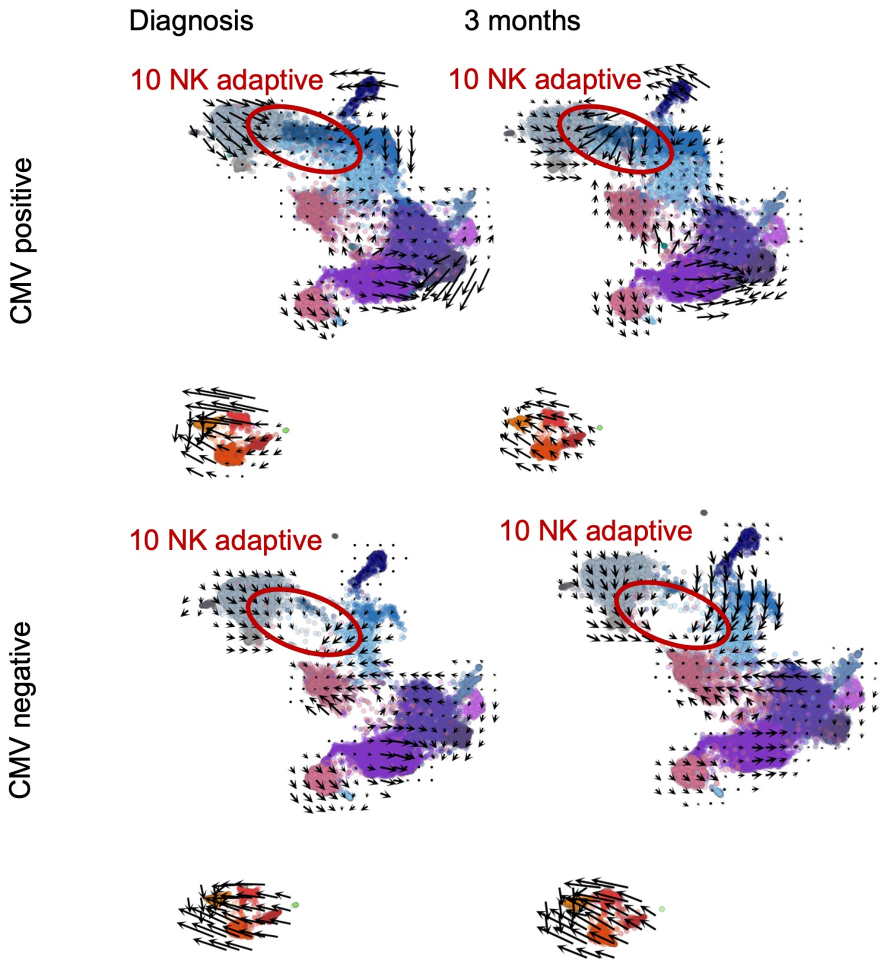

Supplemental Figure 6: RNA turnover analysis

A) UMAP representation of the scRNA-seq samples from CMV seropositive patients (*n*=4) and CMV seronegative patients (*n*=2), where superimposed arrows represent the directional flow calculated with Velocyto by comparing the abundances of spliced and unspliced mRNA reads. Arrows are smoothed with Gaussians.

Supplemental Figure 7

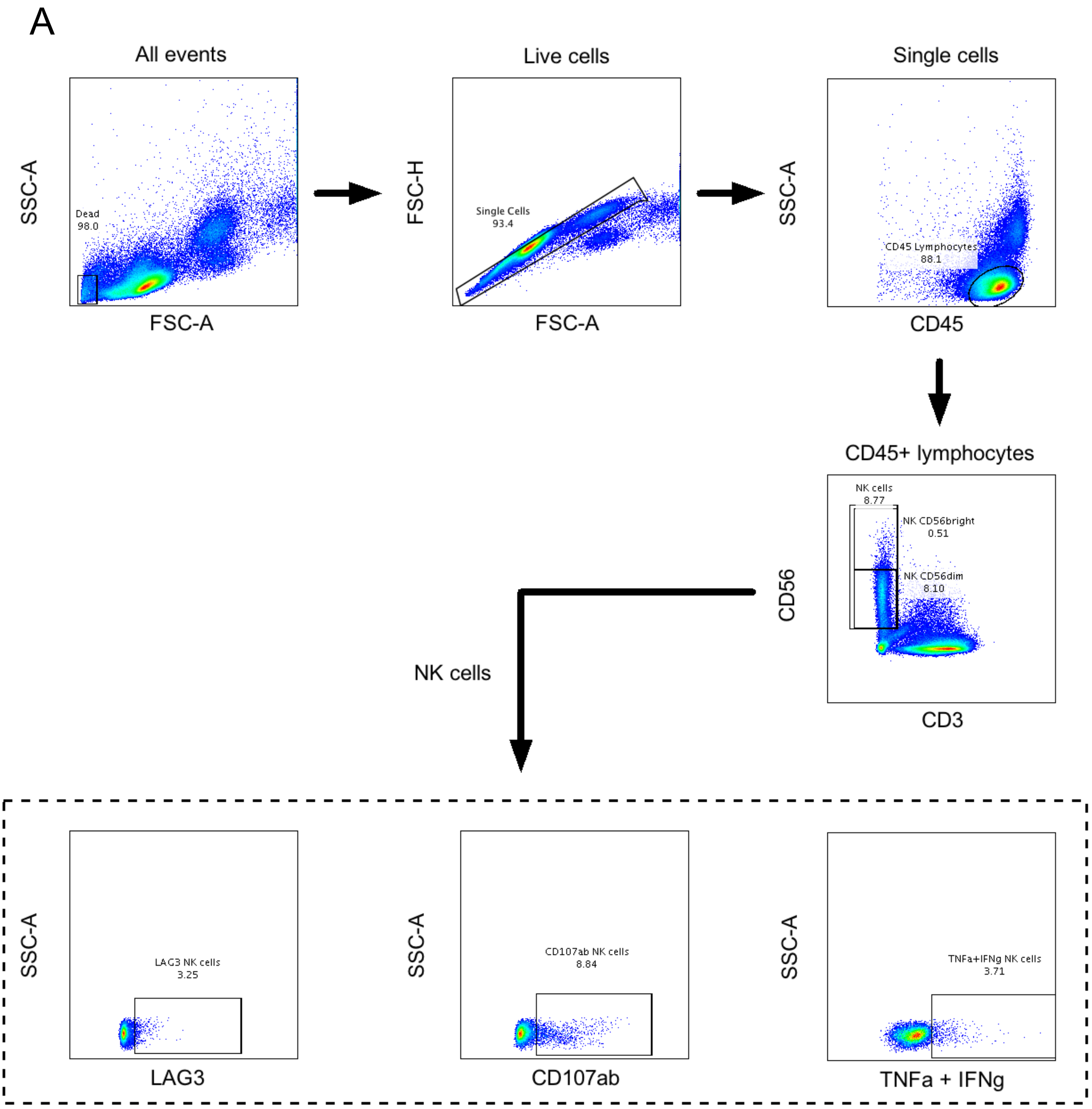

Supplemental Figure 7: The effect of anti-LAG3+anti-PD1 treatment on NK-cells in *ex vivo* analysis

A) The gating strategies for *ex vivo* analysis of primary NK cells stimulated with the CML cell line K562.

Supplemental Figure 8

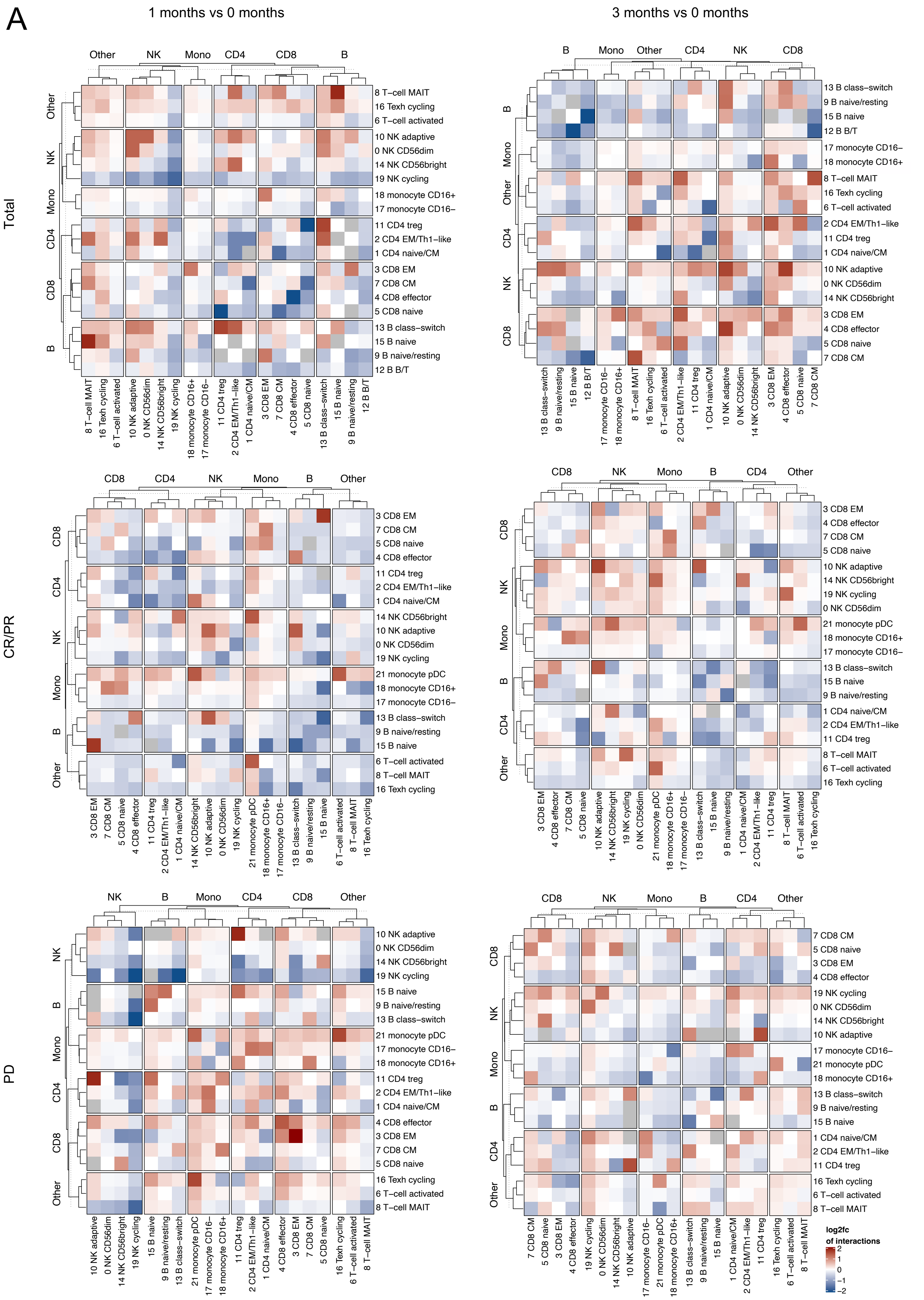

Supplemental Figure 8: Changes in immune cell interactions induced by anti-LAG3+anti-PD1 treatment

A) Heatmaps of the interactome between 1 or 3 months and baseline in all patients (total , *n*=6), patients with response (CR/PR, *n*=3) or patients without a response (PD, *n*=3). The interactome is calculated as a number of receptor-ligand pairs with CellPhoneDB. Red colors indicate growing number of interactions after 1 or 3 months of anti-LAG3+anti-PD1 treatment whereas blue colors indicates a decreasing number of interactions.

Supplemental Figure 9

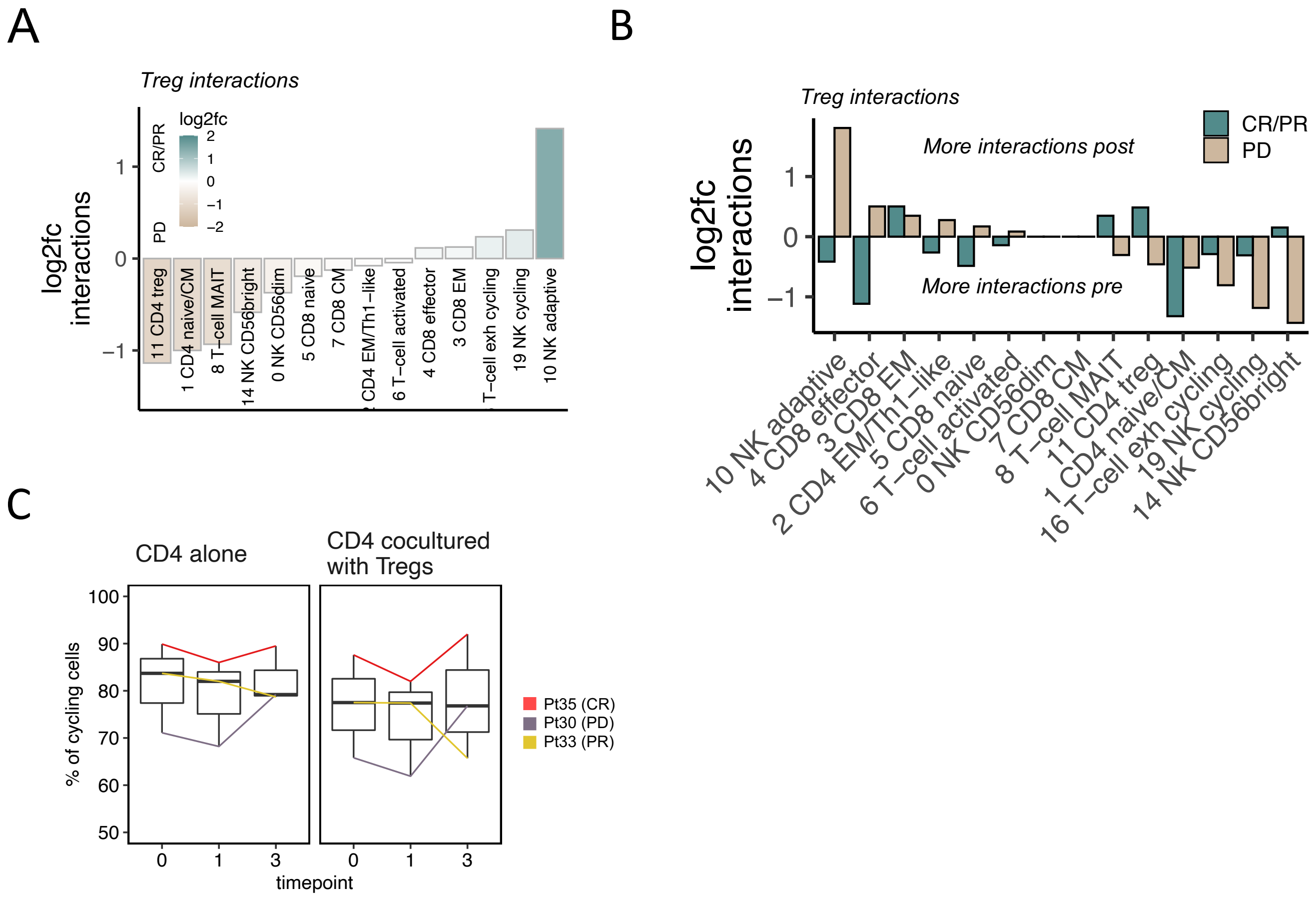

Supplemental Figure 9: Treg suppression capacity decreases following anti-LAG3+anti-PD1 therapy

A) The log2 fold-change of ligand-receptor interactions of Tregs with immune cells in the scRNA-seq between patients with a response (CR/PR) and without a response (PD) at baseline. Statistical test was not calculated due to small sample size.

B) The log2 fold-change of ligand-receptor interactions of Tregs with immune cells after 1 month of anti-LAG3+anti-PD1 therapy in the patients with a response (CR/PR) and without a response (PD). Statistical test was not calculated due to small sample size.

C) Suppression of CD4+ T cell proliferation by Tregs. Samples from 3 patients prior to and after 1 and 3 months of combination treatment were analyzed. The amount of cell proliferation was traced with flow cytometry by dilution of CellTrace Violet dye with presence of CD3/CD28 beads and CD3/CD28 beads and Tregs.

Supplemental Figure 10

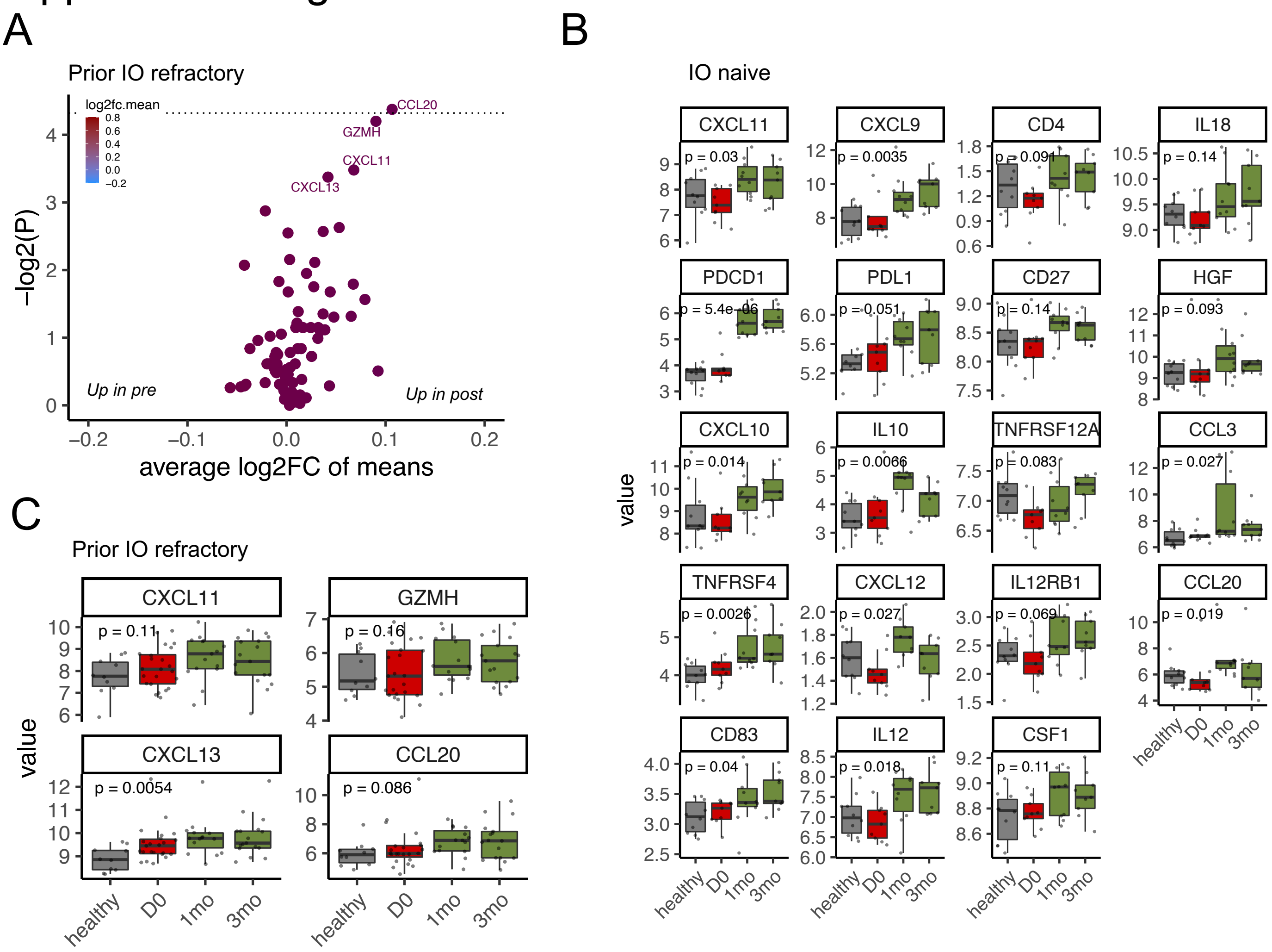

Supplemental Figure 10: Serum protein changes induced by anti-LAG3+anti-PD1 treatment

A) Differentially expressed soluble proteins between before and after therapy in immunotherapy (IO) refractory patients (*n*=24). Proteins with *P*<0.10 are shown, and dashed line denotes *P*=0.05.

B) The proportion of statistically significant (*P*<0.05) serum proteins identified across anti-LAG3+anti-PD1 treatment in immunotherapy (IO) naïve patients (*n*=11).

C) The proportion of (*P*<0.10) serum proteins identified across anti-LAG3+anti-PD1 treatment in immunotherapy refractory patients (*n*=24)

Supplemental Figure 11

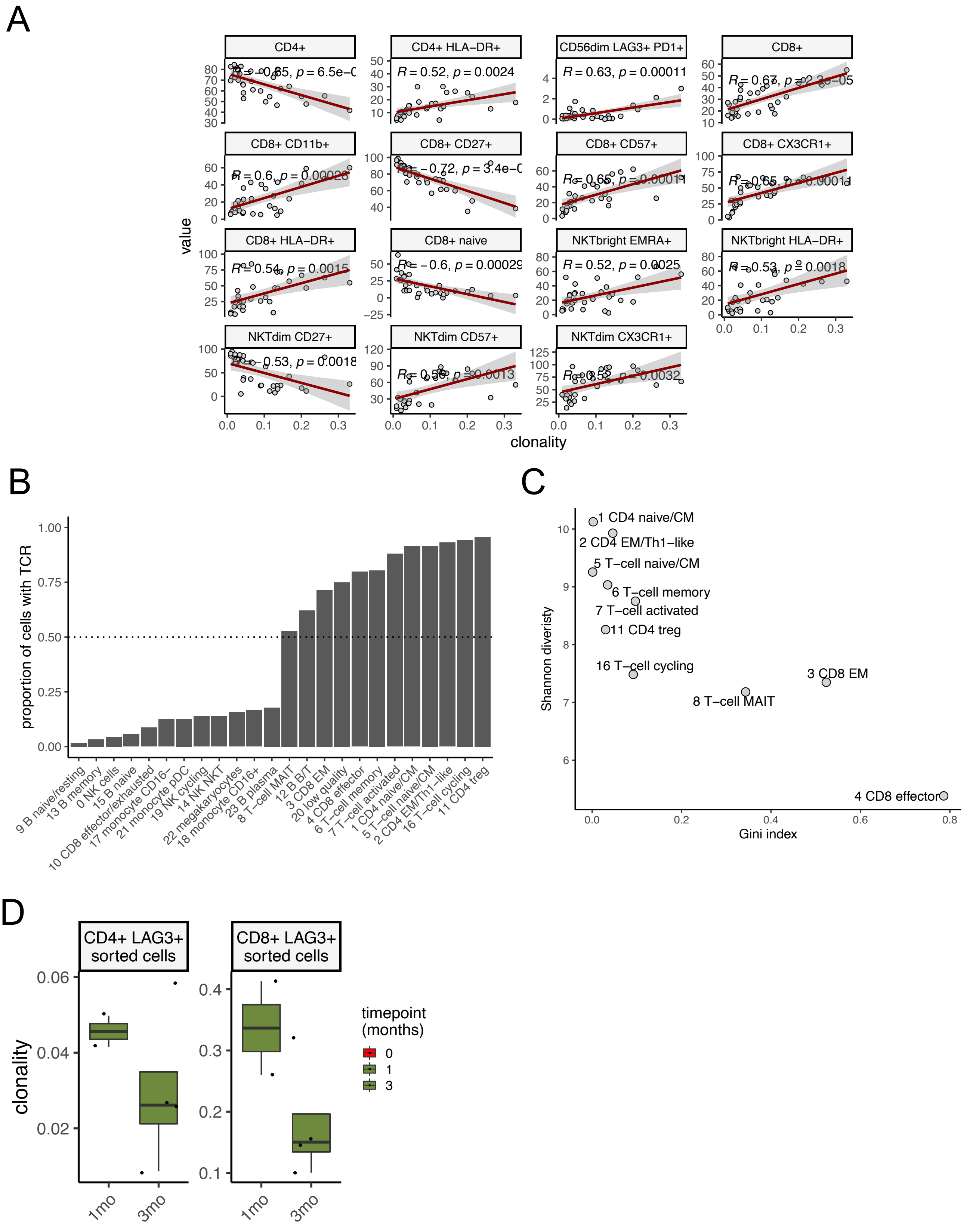

Supplemental Figure 11: T-cell receptor repertoire analysis

A) The association between different immune cell abundancies and overall T-cell clonality in immunotherapy naive and immunotherapy resistant samples from the baseline (*n*=34). The correlation coefficients and *P*-values werer calculated with a Pearson correlation.

B) The proportion of cells with a detected TCRα, TCRβ, or both, in the original clusters seen in Figure 2A.

C) The T-cell receptor repertoire clonality (Gini index, where 1 denotes monoclonal sample) and richeness (Shannon diversity index, where values approaching 0 denote monoclonal samples), in the original clusters seen in Figure 2A.

D) TCR repertoire clonality in LAG3+ CD4+- and LAG3+ CD8+-sorted T cells between 1 and 3 months of anti-LAG3+-anti-PD1 therapy (*n*=6).
